# Sustainable Sound Absorption: A Critical Review of Material Innovation and Geometry-Driven Design

**DOI:** 10.3390/polym18121522

**Published:** 2026-06-18

**Authors:** Faouzia Tayari, Regina Silva, Bruno Godinho, Pedro Pinto, Isabel Cardoso, Tiago Brilhante, Vânia Freitas, Rui Ribeiro, Artur Ferreira, Nuno Gama

**Affiliations:** 1 CICECO—Aveiro Institute of Materials, University of Aveiro, 3810-193 Aveiro, Portugalartur.ferreira@ua.pt (A.F.); 2 Amplitude Acoustics—Acústica e Vibração, Lda., 4470-038 Maia, Portugal; 3 Lightenjin II—Indústria de Iluminação, Lda., 3750-041 Águeda, Portugal

**Keywords:** circular economy, sound absorption, bio-based absorbers, geopolymer-based absorbers, 3D-printed absorbers, recycled absorbers

## Abstract

The transition toward circular economy practices and CO_2_ reduction goals is driving the development of new sound absorption technologies. Traditional absorbers made from mineral wool or foams provide broadband absorption; however, their production is associated with intensive energy consumption and non-renewable resources. This is why the focus has been shifting from the mere substitution of materials to integrated solutions that combine sustainability with structure. This paper reviews recent innovations in sustainable absorbers based on bio-based and recycled materials. The acoustic performance of porous materials depends on such factors such as pore structure, airflow resistivity and geometric parameters such as thickness, multi-layer structure and resonances. At the same time, additive manufacturing (AM) allows creating geometry-controlled absorbers providing advanced acoustic properties. Despite many sustainable absorbers demonstrating sufficient sound absorption properties at medium and high frequencies, their use at low frequencies remains challenging. Additionally, concerns regarding durability, flame retardance, and environmental consistency continue to limit their broader application. Yet, hybrid, multi-material strategies, particularly those combining geopolymer matrices with bio-based or recycled fillers, are identified as a promising route to address these limitations. This review outlines current trends and highlights key challenges and future directions in the design of sustainable sound-absorbing systems.

## 1. Introduction

The importance of noise pollution to the environment and public health is becoming more widely acknowledged [1,2]. According to Halperin [3], exposure to high noise levels is linked to cardiovascular disorders, hearing impairment, stress and disturbed sleep. In addition, prolonged exposure to environmental noise has been shown to negatively affect cognitive performance and overall well-being, making noise control a crucial component of contemporary living spaces, since prolonged exposure to ambient noise dramatically impairs cognitive function and productivity [3,4]. In agreement, Licitra et al. [5] emphasized that regulating noise in urban and industrial settings is now considered a cornerstone of sustainable city planning and occupational safety.

Traditionally, noise control strategies in buildings and transportation depend on two complementary methods: sound insulation (reduce sound transmission between areas) and sound absorption (reduce sound reflections and reverberation within a place). This review primarily focuses on sound-absorbing materials, which typically rely on resonant or porous absorption mechanisms. Glass fibers, mineral wool and polyurethane (PU) foams are examples of traditional absorbers. According to P. Cobo et al. [6], these materials’ porous structures make them useful over a wide frequency range. However, despite their acoustic efficiency, these materials present significant environmental and health concerns. Asdrubali et al. [7] highlighted significant drawbacks: their production requires considerable amounts of energy, they rely on non-renewable feedstocks and disposal typically entails incineration or landfilling, hence intensifying environmental damage. These environmental limitations are increasingly incompatible with sustainability objectives and circular economic principles. Consequently, substantial research has focused on environmentally sustainable alternatives [8,9].

Bio-based materials derived from renewable natural fibers, such as hemp, jute and kenaf, exhibit considerable sound absorption potential due to their fibrous microstructures, which promote effective sound absorption in the mid- to high-frequency range [10,11,12]. Similarly, agricultural residues such as rice husks and sugarcane bagasse have been successfully valorized for sound absorption applications [13,14,15]. The use of such by-products not only provides sound absorption but also reduces environmental impact and material costs in building applications. In addition to bio-based materials, recycled counterparts represent an alternative sustainable pathway [16,17]. Panels manufactured from textile waste exhibit notable sound absorption properties, while recycled polyethylene terephthalate (rPET) fibers can be processed into porous structures that achieve effective noise reduction coefficients [18]. Recycled rubber crumb composites also exhibit both sound absorption and vibration damping [19,20].

**Figure 1:**
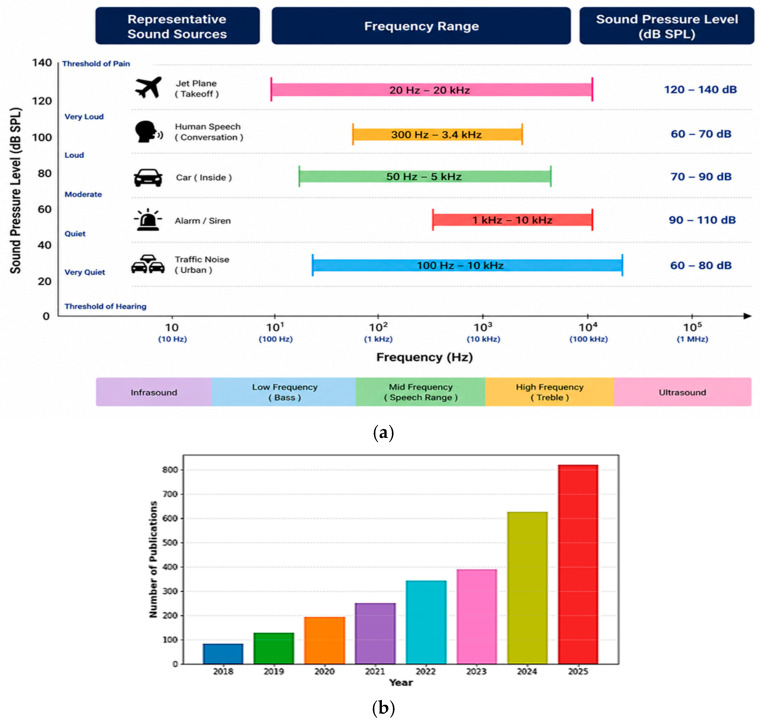
(**a**) Representative sound sources and their typical frequency ranges and sound pressure levels (SPL), compiled from standard acoustics, and (**b**) the annual growth of publications on sustainable acoustic materials; data obtained through a Google Scholar search conducted using the keywords “sustainable sound-absorbing materials” and related terms for the period 2018–2025 (accessed January 2025).

In an effort to understand these material advancements, Figure 1a provides a graphical presentation of the connection between the frequency range, the representative sounds and the associated sound pressure levels.

Environmental and human-induced noises have a wide range of frequencies and intensity levels, ranging from low-frequency traffic noises to high-frequency alarm sounds [21,22]. Yet, no single material is capable of providing optimal acoustic performance across all noise conditions. Consequently, the design of acoustic materials, particularly sustainable ones, must consider key parameters such as porosity, tortuosity, airflow resistivity and thickness, which govern their efficiency across different frequency bands [23,24,25,26]. Apart from bio-based and recycled materials, new classes of inorganic absorbers based on geopolymeric materials have been developed recently [27,28].

Being synthesized from waste products, such as fly ash and slag, geopolymerics provide adjustable porosity in combination with high thermal and flame retardancy as well as excellent mechanical strength [29,30]. This makes them appropriate for application in cases where efficiency and robustness are needed simultaneously. In addition, their use alongside bio-based or recycled fillers allows designing multifunctional hybrid materials.

More recently, additive manufacturing (AM) has introduced a complementary innovation avenue [31,32]. Three-dimensional (3D) printed structures with customized pore geometries can achieve high absorption efficiency within targeted frequency ranges. According to Shanigaram et al. [33,34], high absorption efficiency within specific frequency bands can be attained by 3D-printed structures with customized pore geometries [34,35]. Furthermore, AM allows the incorporation of bio-based or recycled feedstocks [36,37,38], thereby combining material sustainability with geometry-driven performance optimization, offering both environmental benefits and expanded design flexibility.

As illustrated in Figure 1b, the number of publications on sustainable acoustic materials has increased steadily from 2018 to 2025, reflecting growing scientific and industrial interest in environmentally responsible sound absorption technologies. The selected period (2018–2025) corresponds to the rapid expansion of research on circular economy strategies, AM technologies and multifunctional sustainable acoustic materials. This trend highlights the evolution of the field toward sustainability-driven innovation. It also underscores the urgent need to develop viable substitutes for traditional absorbers within low-carbon construction strategies [39]. Despite substantial progress, the field remains fragmented. Many studies report promising sound absorption coefficients but provide limited assessment of life cycle impact, durability under environmental exposure, scalability of production or regulatory compliance [8,40,41]. Moreover, sustainable acoustic materials are often evaluated primarily through intrinsic porosity and airflow resistivity without integrating structural optimization or resource efficiency considerations [24,25].

Consequently, the balance between sound absorption and environmental benefit is not yet comprehensively established. A critical transition is now underway: sustainable sound absorption is evolving from simple material substitution toward integrated sustainability engineering. In bio-based and recycled systems, absorption is largely governed by material morphology and fiber architecture [12,42]. In contrast, AM systems enable geometry-driven acoustic control, potentially reducing material usage while targeting specific frequency ranges [43,44]. This convergence of material innovation and structural design represents a key opportunity to enhance performance while minimizing environmental impact.

Although numerous reviews have examined specific categories of sustainable acoustic materials, such as natural fibers, recycled materials, geopolymers and acoustic metamaterials, a comprehensive assessment that integrates these approaches within a unified sustainability framework remains limited. This review addresses this gap by providing a critical and comparative analysis of four emerging classes of sustainable sound-absorbing systems: bio-based materials, recycled materials, geopolymer-based materials, and architected structures enabled by AM. Unlike previous reviews that focus primarily on material composition, this work examines both material-driven and geometry-driven mechanisms governing sound absorption. Particular emphasis is placed on the relationships among pore structure, airflow resistivity, geometric design, acoustic performance, durability, life cycle considerations and environmental sustainability. Furthermore, the review highlights recent advances in hybrid and multifunctional systems that combine sustainable materials with engineered architectures to enhance sound absorption, resource efficiency and environmental compatibility. By bridging developments in materials science, acoustic engineering, and advanced manufacturing, the review provides a comprehensive perspective on current achievements, existing challenges and future research directions for next-generation sustainable sound-absorbing technologies. By placing sound absorption materials within the broader framework of sustainable construction and circular economy principles, this review aims to clarify both the environmental potential and the practical limitations of current technologies. Ultimately, sustainable sound absorption should be regarded not merely as an alternative material approach but as a fundamental component of low-carbon, resource-efficient building systems.

## 2. Sound Material Mechanisms

Sound absorption refers to the dissipation of acoustic energy, primarily through its conversion into heat when sound waves interact with a material surface. The effectiveness of sound absorption is influenced by the material’s overall structure (including porosity, tortuosity and pore size) and the frequency of the incident sound [45,46,47]. An understanding of these phenomena is essential for creating sustainable [15] and high-performance sound absorber materials [11]. Other than thermal and viscous dissipation within porous materials, the incoming sound waves may excite molecular chain movement in the materials, resulting in energy losses [48,49]. Additionally, when the absorption is dependent on the properties of fillers, matrix thickness and structure, a combination of structures designed to dissipate energy in multiple ways can significantly enhance acoustic performance [50]. Overall, the absorption characteristics of the global material emerge from the interactions between the various systems.

### 2.1. Basic Mechanisms of Sound Absorption

Sound absorption in porous materials is governed by multiple physical processes rather than a single phenomenon [45]. Among these, three dominant mechanisms are generally recognized as responsible for the dissipation of sound energy within such structures [51]:

Viscous losses occur when incident sound waves induce oscillatory airflow through interconnected pores, such as in fibrous or foam-based materials [52]. Friction between air particles and pore walls converts part of the sound energy into heat, making this mechanism particularly effective at mid- to high-frequency ranges [52,53]. Thermal losses arise from alternating the compression and rarefaction of air within the pores. The temperature gradients generated between the oscillating air and the solid matrix promote periodic heat exchange, contributing to additional acoustic energy dissipation [53,54]. Resonant absorption is observed in structures such as thin panels and Helmholtz resonators [55]. At specific frequencies, incident sound waves excite structural or cavity resonances, resulting in significantly enhanced sound absorption within narrow frequency bands, especially at low frequencies [55,56,57].

Overall, the effectiveness of porous absorbers is strongly frequency-dependent. At medium and high frequencies, sound waves interact efficiently with pore walls and fiber surfaces, producing viscous and thermal losses that convert sound energy into heat. However, low-frequency sound waves possess much longer wavelengths and therefore interact less effectively with thin porous structures. Consequently, achieving significant low-frequency absorption typically requires greater material thickness, deeper air cavities, resonant elements, or engineered architectures such as metamaterials. This fundamental physical constraint explains why many sustainable acoustic materials exhibit stronger absorption at medium and high frequencies than at low frequencies.

### 2.2. Principles of Sound Absorption

The effectiveness of sound-absorbing materials is commonly characterized using parameters that describe how efficiently they dissipate acoustic energy [58]. The sound absorption coefficient α(f) (or SAC—sound absorption coefficient) is related to the reflection coefficient R(f) as defined by Allard and Atalla (2009) [51] being expressed mathematically as:

(1) $$\alpha \left(f\right)=1-{\left|R\left(f\right)\right|}^{2}$$

Since the phase component of R(f) is omitted, the α(f) provides less detailed information compared to impedance or the full reflection coefficient. Nevertheless, it is widely used in architectural acoustics, where this simplification proves to be useful. In such cases, it can be reformulated as:

(2) $$\alpha \left(f\right)=1-\frac{E^{\prime }\left(f\right)}{E\left(f\right)}$$

where E(f) is the incident acoustic energy and E′(f) is the reflected acoustic energy. Thus, α(f) ranges from 0 to 1, where α = 0 indicates total reflection (no absorption) and α = 1 indicates total absorption (no reflection). In practice, intermediate values describe the proportion of energy absorbed by the material surface, making α(f) a key parameter in architectural acoustics and material characterization [51].

For applied assessment in building and architectural acoustics, the noise reduction coefficient (NRC) is often used. It provides a single-number rating of material performance, calculated as the average of α(f) at four standard frequencies, 250, 500, 1000 and 2000 Hz [59], according to:

(3) $$NRC=\frac{\alpha_{250}+\alpha_{500}+\alpha_{1000}+\alpha_{2000}}{4}$$

where $\alpha_{250}$, $\alpha_{500}$, $\alpha_{1000}$ and $\alpha_{2000}$ represent the sound absorption coefficients at the corresponding frequencies.

Another important parameter in room acoustics is the reverberation time (RT60) [60], which represents the time required for the sound level to decay by 60 dB after the source is turned off [61]. The classical Sabine formula relates RT60 to the room volume and total absorption as [62]:

(4) $${RT}_{60}=\frac{0.161V}{A}$$

where V is the room volume (m^3^) and A is the equivalent absorption area (m^2^), calculated as the sum of the products of each material’s surface area and its absorption coefficient. The Sabine equation provides accurate predictions primarily in rooms with relatively diffuse sound fields and moderate absorption levels; however, its accuracy decreases in spaces with highly non-uniform sound distributions, strong directional reflections or very high absorption, where alternative models such as the Eyring or Millington–Sette formulations may provide more reliable predictions.

These parameters α(f), NRC and RT60 are essential for evaluating material performance and designing acoustically optimized spaces [62,63].

### 2.3. Types of Sound Absorbers

**Figure 2:**
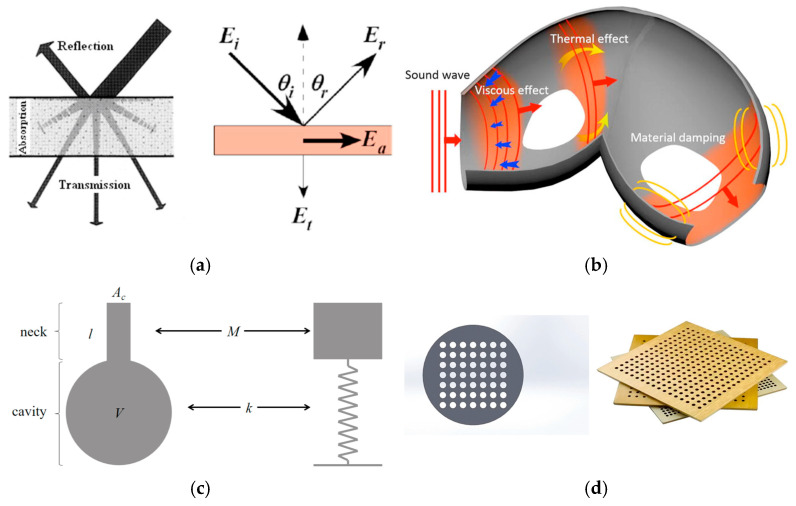
Schematic of (**a**) illustration of sound absorption mechanisms in porous materials (reproduced with permission from Elsevier B.V., 2016) [71], Elsevier, 2017; (**b**) energy dissipation pathways in porous sound-absorbing materials (reproduced with permission from the American Institute of Physics, 2017) [68], AIP, 2017; (**c**) Helmholtz resonator (HR) and its equivalent mass–spring model, reproduced with permission from [56], MDPI, 2021 and (**d**) micro-perforated panel (MPP) structures, reproduced with permission from [70], Springer, 2025.

Sound absorbers can be classified according to their sound energy dissipation mechanisms and effective frequency ranges, mainly into porous absorbers [64], membrane (panel) absorbers and resonant systems such as Helmholtz resonators [65,66]. Porous absorbers rely on interconnected pore networks that allow sound waves to penetrate the material [67]. When sound waves strike a porous surface, the incident energy is divided into reflection, absorption and transmission (as illustrated in Figure 2a). The absorbed energy propagates through the pores and is dissipated as mechanical and thermal energy through viscous and thermal interactions, as well as through resonance of the pore walls (Figure 2b) [68]. While porous absorbers are efficient at mid-to-high frequencies, their performance at low frequencies is limited. This motivates the use of resonant systems like Helmholtz resonators which are used to further improve low-frequency absorption and accomplish frequency-selective control [56].

In classical theory, a Helmholtz resonator can be modeled as an equivalent mass–spring system, as shown in Figure 2c. The neck acts as the connecting pathway between the cavity and the external environment and its geometric parameters are key factors in determining the resonance frequency [55]. Membrane (panel) absorbers are made up of thin, flexible panels that are supported by an air cavity [69]. The sound waves that enter the cavity cause the panels to vibrate, transforming the sound energy into mechanical motion that is then released [69]. Their mass–spring system, which is controlled by the panel mass, stiffness and cavity depth, allows absorption to be expanded and pushed toward lower frequencies by adding porous materials [70]. A micro-perforated panel is shown schematically and in real view in Figure 2d.

### 2.4. Influence of Material Properties

The acoustic response of a material depends on several intrinsic parameters that govern how sound waves interact with its internal structure [72]. Among these, porosity [73], flow resistivity [74], tortuosity and thickness are the most influential factors in determining the overall absorption performance [75].

Porosity refers to the fraction of a material’s total volume that is occupied by the fluid (air) phase within its pores. If $V_{t}$ represents the total volume of the porous medium [51], $V_{s}$ and $V_{f}$ denote the volumes of the solid and fluid phases respectively, then porosity can be expressed as:

(5) $$\phi =\frac{V_{f}}{V_{t}}=1-\frac{V_{S}}{V_{t}}$$

It represents the degree to which a material contains interconnected voids that allow air movement and sound wave penetration. For materials with high porosity, the relationship can also be approximated in terms of densities:

(6) $$\phi =1-\frac{\rho_{s}}{\rho_{m}}$$

where $\rho_{s}$ is the solid phase or frame density and $\rho_{m}$ the solid phase material density.

Flow resistivity represents the resistance encountered by air as it moves through the interconnected pores of a material. It is defined as the ratio between the steady airflow pressure difference across the material and the corresponding velocity of the airflow, measured in Pascal-seconds per square meter (Pa·s/m^2^). Mathematically, it can be expressed as [51]:

(7) $$\sigma =\frac{∆P}{v\;d}$$

where ΔP is the pressure drop across a sample of thickness d and v is the average particle velocity of air through the pores.

Tortuosity is a dimensionless structural parameter that describes how the internal pore geometry of a material affects the macroscopic flow of air through it and relates directly to the material’s acoustic performance [75,76]. Experimentally, tortuosity can be determined by saturating a non-conductive porous sample with a conductive fluid and measuring its electrical resistivity [77]. If $R_{m}$ and $R_{f}$ represent the resistances of the saturated material and the fluid, tortuosity $\left(\alpha_{\infty }\right)$ can be expressed as:

(8) $$\alpha_{\infty }=\phi \frac{R_{m}}{R_{f}}$$

The thickness (d) of a material significantly influences its ability to attenuate sound energy, particularly at low frequencies. ALRahman et al. [12] reported that for any porous structure the SAC increases proportionally with material thickness. Similarly, other researchers have shown that at lower frequencies, the effectiveness of a material in reducing sound transmission is directly linked to its thickness. Increasing the thickness enhances overall absorption and shifts the absorption peak toward lower frequencies [78]. This improvement results from a prolonged dissipative process of viscous and thermal interactions between air and the absorbing material, leading to enhanced absorption with greater thickness.

### 2.5. Sound Absorption Measurement Using the Impedance Tube Method

**Figure 3:**
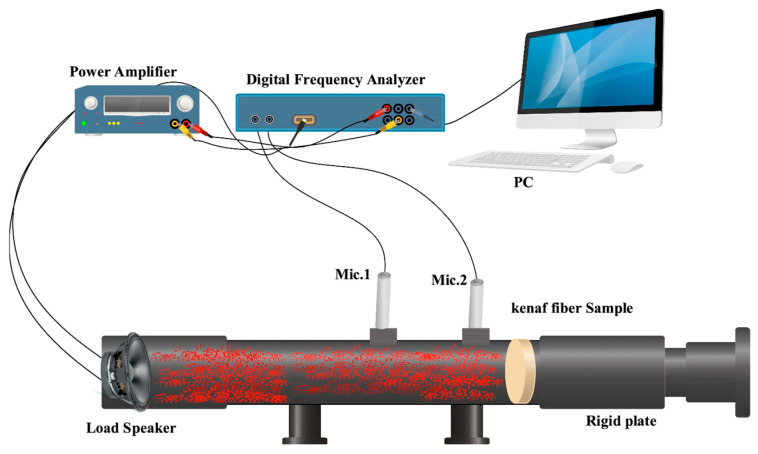
A schematic of the impedance tube system, reproduced with permission from [79], Springer, 2021.

The equipment used to determine the SAC at normal incidence is shown in Figure 3. A frequency analyzer, two microphones and a loudspeaker make up the impedance tube system. The loudspeaker at one end of the tube generates a sound signal that passes through the tube and hits the surface of the sample. To ensure even testing conditions the sample is placed into a holder. Two microphones located at different positions along the impedance tube recorded the reflected sound signals. The SAC is then calculated using specific analysis software.

## 3. Bio-Based Sound-Absorbing Materials

**Figure 4:**
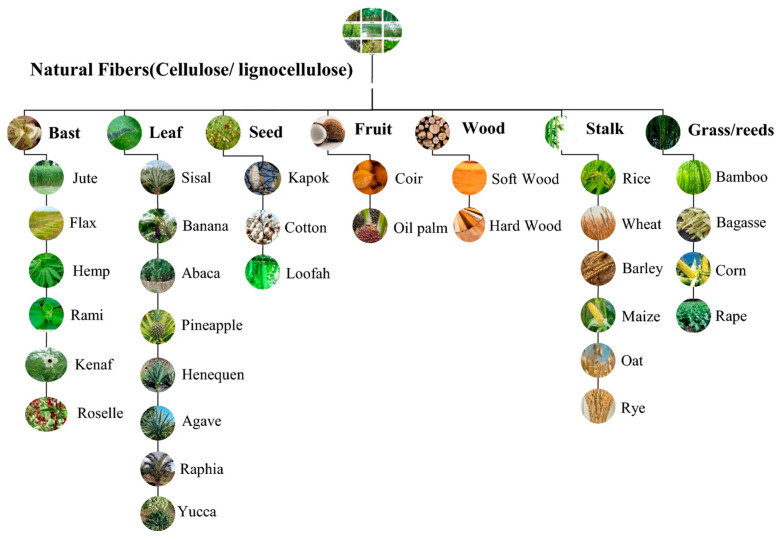
Classification of natural fibers used as sound-absorbing materials into three categories: animal, plant and mineral; reproduced with permission from [79], Springer, 2021.

Bio-based sound-absorbing materials are gaining increasing attention due to the need to address current environmental issues and the need to avoid the use of synthetic and non-renewable materials. These materials are produced using renewable sources such as natural fibers [80], agricultural residues or bio-composites which offer sound absorption and environmental sustainability. The effectiveness of the materials to absorb sound energy is due to their fibrous microstructure [81], low density and high porosity, which provide the dissipation of sound energy [82]. Figure 4 illustrates natural fibers as sound-absorbing materials.

### 3.1. Natural Fibers

Natural fibers including kenaf [80], jute [83,84], flax [83], hemp [85,86,87], coir [88,89], sisal [90] and woods [91] have attracted significant interest due to their abundance, low cost [92] and biodegradability [92]. For example, kenaf fibers have been extensively investigated for acoustic applications due to their low density, high porosity and fibrous microstructure which promote viscous and thermal losses during sound propagation [80]. Natural fibrous materials exhibit high sound absorption performance, particularly at medium and high frequencies. Yet, experimental investigations by Berardi et al. [11] reveal that natural fiber absorbers have limited efficiency in the low-frequency range (125–250 Hz) as shown in Table 1. Please note that the reported values originate from different studies and should therefore be interpreted as indicative rather than directly comparable, especially considering differences in material thickness and testing conditions.

Kenaf panels, with high thickness and density, can achieve SAC near unity in the 1000 Hz to 2000 Hz range, resulting in NRC values up to 0.70. This is echoed in materials like sheep wool and coconut fibers where airflow resistivity, fiber morphology and panel thickness largely influence sound absorption response. Materials with open and tortuous fiber networks, such as sheep wool and kenaf, outperform denser systems, indicating that interconnected porosity is crucial.

**Figure 5:**
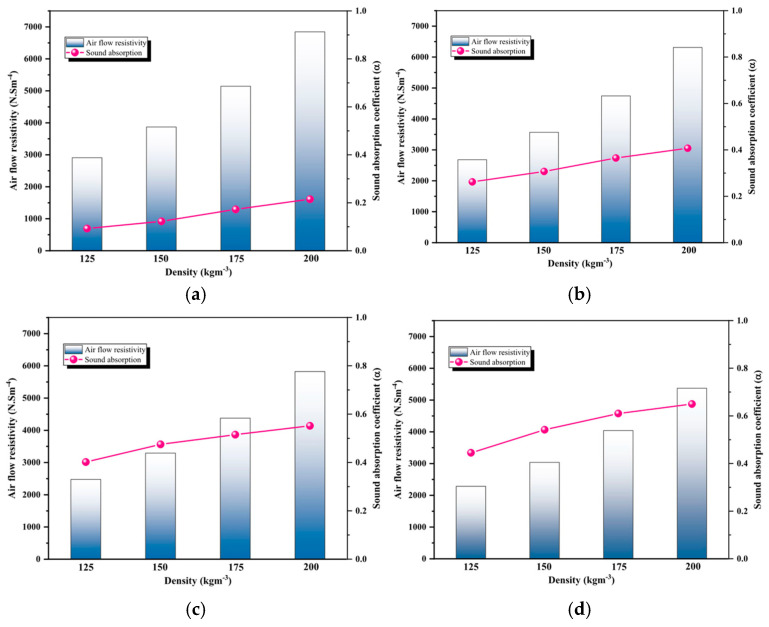
The SACs of the samples with different thickness (**a** 15, **b** 25, **c** 35, **d** 45 mm) of kenaf fibers with different densities and airflow resistivity; reproduced with permission from [79], Springer, 2021.

While natural fibers excel in mid-frequency absorption, their low-frequency performance is largely thickness-dependent. Taban et al. [79] reported that increasing the thickness and bulk density of kenaf fiber samples significantly enhances sound absorption performance. The improvement is mainly attributed to higher fiber packing which increases airflow resistance and creates a more tortuous path for sound waves. This leads to greater viscous friction and consequently, higher sound energy dissipation. Their findings, illustrated in Figure 5a–d, show that samples with greater thickness (15–45 mm) and density exhibit higher SAC.

Natural fiber absorbers offer a sustainable alternative to synthetic materials, with their sound absorption performance being highly dependent on their structure [12,80]. Factors like thickness, bulk density, airflow resistivity and fiber morphology influence their sound dissipation [25,93,94]. Nevertheless, their low-frequency limitation remains thickness-controlled rather than material-specific, often requiring structural modification or hybrid integration [26,68]. Therefore, although natural fibers are environmentally advantageous, their effective application requires careful microstructural optimization rather than simple material substitution. Additionally, agricultural residues are inherently heterogeneous materials, whose physical and chemical properties can vary depending on plant species, cultivation conditions, geographic origin, harvesting season, moisture content, particle size distribution and processing history. These variations can directly affect key acoustic parameters such as porosity, tortuosity, airflow resistivity, density and, consequently, sound absorption performance.

### 3.2. Agricultural Residues

**Figure 6:**
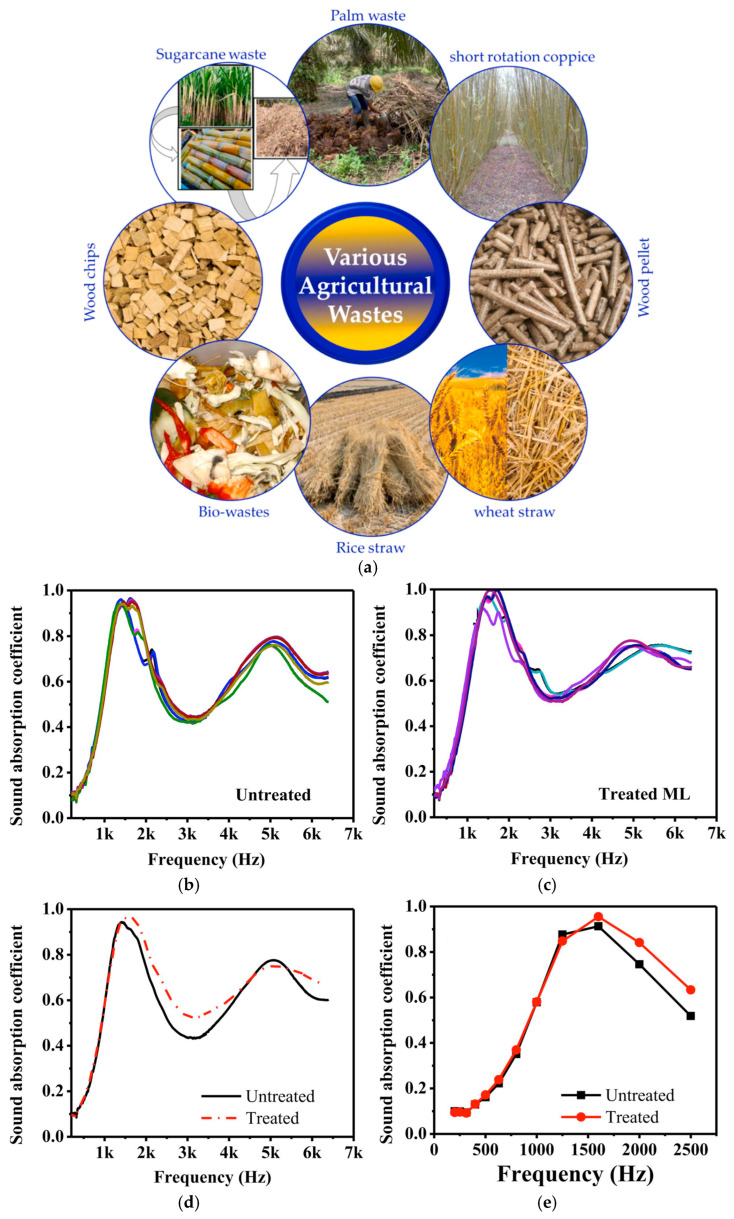
Various agricultural residues as sources for sound absorbers (**a**), reproduced with permission from [102], Elsevier, 2023. SAC results of (**b**) untreated leaf; (**c**) treated leaf; (**d**) comparison between untreated and treated leaf waste; and (**e**) comparative performance in 1/3 octave bands, reproduced with permission from [103], Elsevier, 2024.

The valorization of agricultural residues presents a promising circular economy opportunity for converting agro-industrial waste into cheap sound absorbers [95,96,97]. The inherent porosity and fiber structure of rice husk [13,14], sugarcane bagasse [15,98], palm [95], wood chips [99], wheat straw [100] and maple leaf waste enhance thermoviscous dissipation within the interconnected pore structure [53,101]. Additionally, the low density and irregular particle size of agricultural residues increase tortuous paths for airflow, a key factor for mid-frequency sound absorption [95,100]. Figure 6a illustrates typical examples of agricultural residues and sound absorption panels derived from them, highlighting their porous morphology and their suitability for sustainable noise control applications [102].

In addition, experimental studies have shown that microstructural modification of agricultural wastes significantly affects sound absorption efficiency. For example, modification of maple leaf waste through chemical treatments enhances pore formation and surface area, leading to improvements in sound absorption coefficients. Haradhan Kolya et al. [103] studied untreated (control) and treated maple leaf waste samples. SAC results are presented for the control specimens in Figure 6b and treated specimens in Figure 6c. Figure 6d provides a direct comparison between the two groups, while Figure 6e provides the 1/3 octave band analysis to show acoustic performance [103]. The results prove that sound absorption efficiency is not only dependent on the type of agricultural residue but also on pore structure and airflow resistivity.

On the other hand, the sound absorption of agricultural residue-based absorbers is highly dependent on particle size and density. Larger particles are expected to increase pore connectivity and viscous dissipation but they may compromise the mechanical properties of the absorber [94,95,100]. In contrast, the densification of agricultural residues, especially through the use of bio-based adhesives such as starch and soy protein, may reduce pore channel size, increase density and compromise airflow, leading to a reduction in acoustic efficiency. Another limitation of agricultural residue-based sound absorbers is that they are highly susceptible to moisture and long-term durability challenges [104,105]. The hygroscopic nature of lignocellulosic agricultural residues may also compromise airflow resistivity, mechanical stability and sound absorption performance in real-time environments [25,97,106]. Therefore, the main challenge in agricultural residue-based acoustic absorbers is to find a compromise between porosity, mechanical integrity and environmental durability. Conclusively, agricultural residues are promising candidates for acoustic applications but their sound absorption performance depends on microstructural modification and not merely on density and compaction of agricultural wastes.

### 3.3. Mycelium and Other Bio-Composites

Mycelium-based composites represent an emerging category of bio-derived acoustic materials where mycelium acts as a natural adhesive [107,108], converting loose lignocellulosic materials into cohesive and porous materials [106]. Unlike conventional natural fiber materials, mycelium materials are biologically engineered, allowing control of their microstructural arrangement.

**Figure 7:**
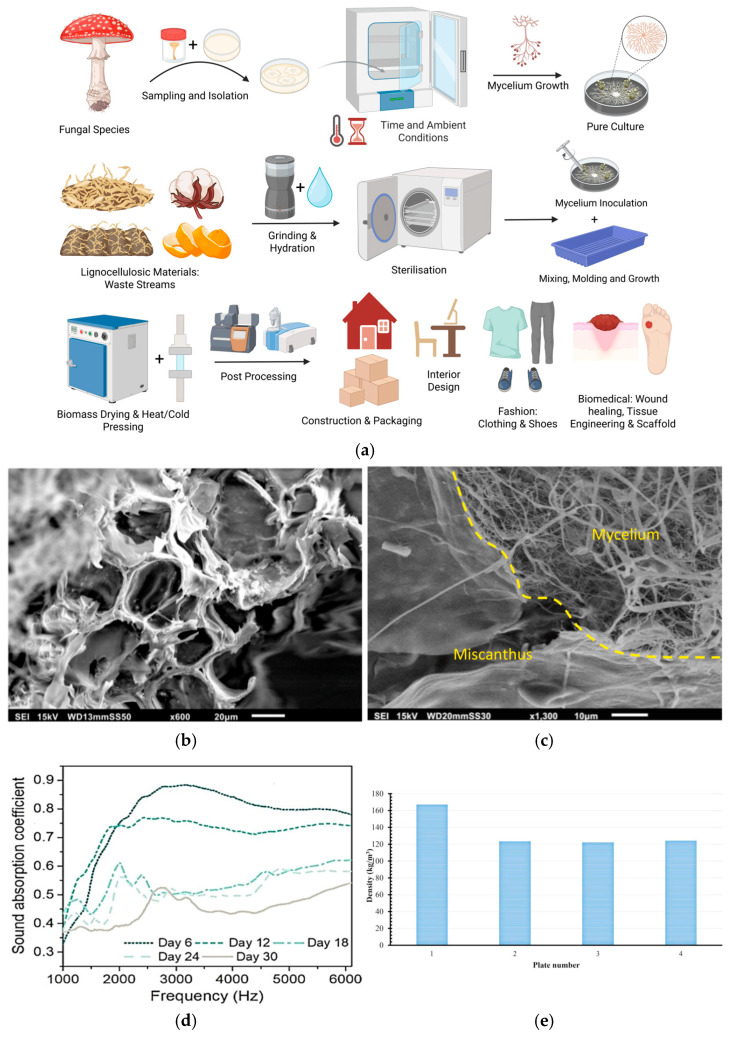
(**a**) A schematic of the fabrication process of mycelium-based composites, reproduced with permission from [109], MDPI, 2025; (**b**) a SEM image showing the interconnected hyphal network and Miscanthus fibers, reproduced with permission from [110], Elsevier, 2021; (**c**) a high-magnification view of hyphae growing around fibers forming a porous structure, reproduced with permission from [110], Elsevier, 2021; (**d**) SAC showing the influence of incubation time, reproduced with permission from [109], MDPI, 2025; and (**e**) the density range of the produced mycelium-based acoustic panels, reproduced with permission from [110], Elsevier, 2021.

Mycelium-based composites are produced by growing fungal species on lignocellulosic substrates where hyphae act as natural binders to form lightweight, porous structures with good sound absorption. As illustrated in Figure 7a, the process involves sterilization, inoculation, incubation (10 days) and drying, resulting in a rigid bio-foam with interconnected pores.

The sound absorption performance of mycelium materials is determined by the formation of interconnected networks of mycelium which increase their tortuosity, thus affecting airflow resistivity. SEM images of mycelium materials have always shown the presence of interconnected filaments, which bridge the particles, thus creating porosity (Figure 7c,d). The porosity enhances thermoviscous dissipation, thus increasing sound absorption. The efficiency of sound absorption, however, relies on growth conditions such as type of fungus, substrate, growth period and drying process [109,111,112].

From experimental investigations, sound absorption is strongly influenced by incubation time, which governs microstructure development and airflow resistivity [110]. Zahra Parhizi et al. [109] reported SAC values above 0.5, reaching 0.87 at 2800 Hz after six days of growth (Figure 7d), demonstrating the link between growth conditions, microstructure and sound absorption efficiency.

As illustrated in Figure 7e, densities of 122.5–167.3 kg/m^3^ enhance sound dissipation while reducing structural load. Despite the promising sound absorption properties of mycelium-based materials, their low-frequency performance, similar to other porous bio-derived materials, is constrained by material thickness, with no inherent biological mechanisms contributing to sound absorption [111,113]. Although mycelium materials have low density, are biodegradable and require less non-biological materials [106,110,114], they still face several challenges, including:

- Lack of understanding of strain–microstructure–acoustic correlations;
- Heterogeneity due to substrate effects;
- Lack of information on long-term durability and stability in humid environments;
- Fire resistance;
- Lack of information on scaling up production from laboratory-scale to industrial production.

Although mycelium materials offer a biologically innovative solution in the bio-derived materials field, their transition from laboratory prototypes to building materials requires the optimization of growth parameters, durability and hybridization with other acoustic materials to improve their low-frequency sound absorption [106,110,112,114].

Table 2 demonstrates that, despite differences in structural formation mechanisms (mechanical compaction in natural fibers and agricultural residues versus biological self-assembly in mycelium composites), these materials share underlying similarities in their resulting porous architectures [11,95,115]. All bio-based absorbers remain fundamentally governed by porosity [23,116], tortuosity and airflow resistivity [24,25]. Although mycelium systems provide biologically tunable architectures and reduced dependence on synthetic binders [106,110], none of the categories intrinsically overcome low-frequency absorption limitations without increased thickness, geometric modification or hybrid integration.

## 4. Recycled Materials for Sound Absorption

In the field of acoustics, the utilization of post-consumer and post-industrial waste has become an important strategy for developing sustainable materials [16,20,48]. Recycled resources can be transformed into low-cost sound-absorbing materials that often provide sound absorption performance comparable to conventional synthetic products [129,130,131], while lowering embodied energy and minimizing landfill waste [37,48].

**Figure 8:**
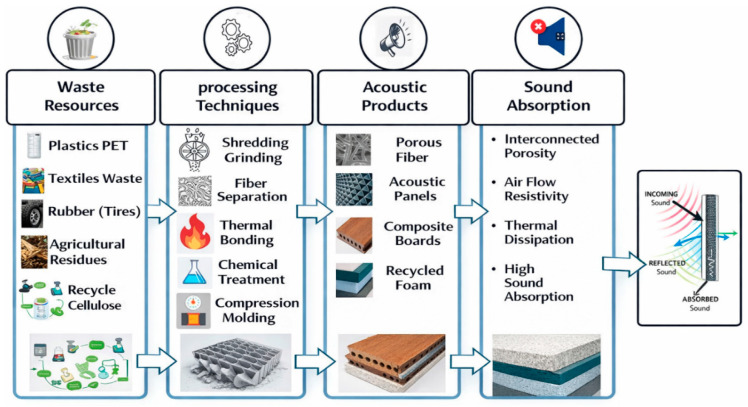
Schematic of recycled materials and their conversion into sound-absorbing acoustic systems.

An overview of the main recycled material streams used for acoustic applications is presented in Figure 8, highlighting their processing routes and conversion into porous sound-absorbing systems. Recycled textiles [132], recycled polymers and recycled rubber-based composite systems are some of the most extensively studied categories [20,133,134]. They all have unique microstructural characteristics that affect their acoustic behavior [20,130,132].

### 4.1. Recycled Textiles

**Figure 9:**
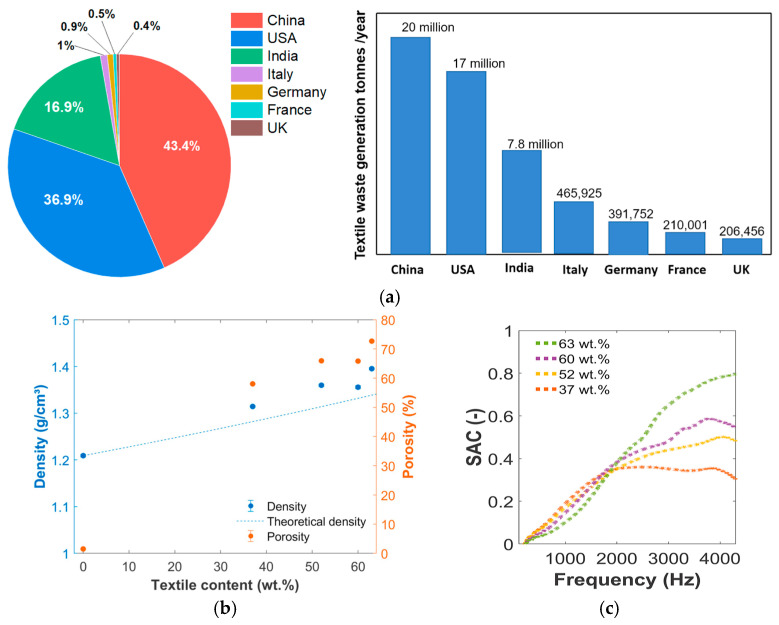
(**a**): Global textile waste and annual generation by country (reproduced with permission from BusinessWaste.co.uk [138], SAGE, 2025); (**b**) variation in density and porosity with textile content, reproduced with permission from [139], Elsevier, 2025; and (**c**) SAC with variation in textile content, reproduced with permission from [139], Elsevier, 2025.

Textile production is one of the leading waste producers around the world [135,136], with about 92 million tons of textile waste produced annually [137]. Additionally, forecasts indicate that this number is expected to increase sharply by 2030. Leading producers of waste in this area include China, the USA, India and some European countries, as illustrated in Figure 9a.

The textile waste flow poses challenges and opportunities alike regarding their acoustics application. Recycled textile fibers, including cotton [140,141], wool [142], polyester blends and industrial offcuts are increasingly studied as sound absorbers [140]. Their effectiveness comes from high porosity [84], irregular fiber orientation and adjustable compaction which promote viscous and thermal energy dissipation [143]. Unlike mineral wool [98,144], recycled textiles can be mechanically processed to control density and airflow resistivity, allowing tuning of their acoustic response [138,145]. Ferreira Junior et al. [139] showed that polyester-based textile panels exhibit good mid- to high-frequency absorption, especially when textile content increases.

As illustrated in Figure 9b, higher fiber fractions lead to greater porosity and improved SAC, reaching values close to 0.8 at high frequencies. This confirms that fiber packing and internal pore structure are more influential than fiber type alone [146,147]. This structural change directly impacts sound absorption properties. Parameters like textile content, thickness and compaction significantly affect the SAC, particularly at medium and high frequencies. Figure 9c shows that higher textile fractions improve sound absorption performance, with the 63 wt.% sample reaching SAC values close to 0.8 at high frequencies. These findings confirm that recycled textile composites are both environmentally sustainable and technically suitable for sound insulation in buildings. While the acoustic properties of recycled textile panels appear to be quite promising, particularly at mid- and high-frequency ranges [148], their potential widespread use is unclear. One of the challenges faced is the inconsistency of waste textiles available, thus making the process difficult to replicate and reliable for sustained use [149]. Furthermore, research studies have been primarily concentrated on conducting tests using impedance tubes, with minimal investigations on aspects such as fire resistance [125,126], aging and moisture resistance during actual usage [105,150].

### 4.2. Recycled Polymers

**Figure 10:**
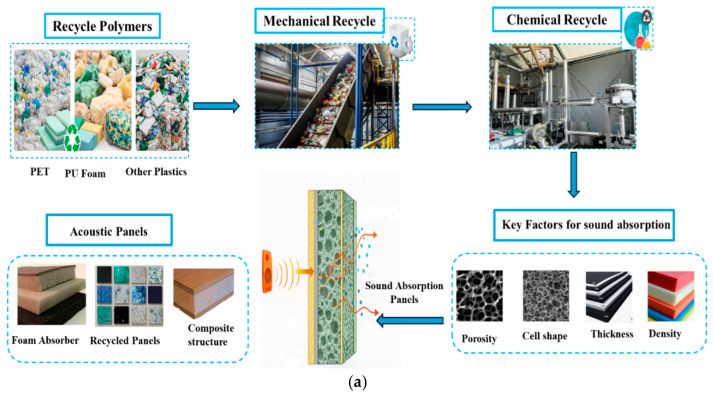

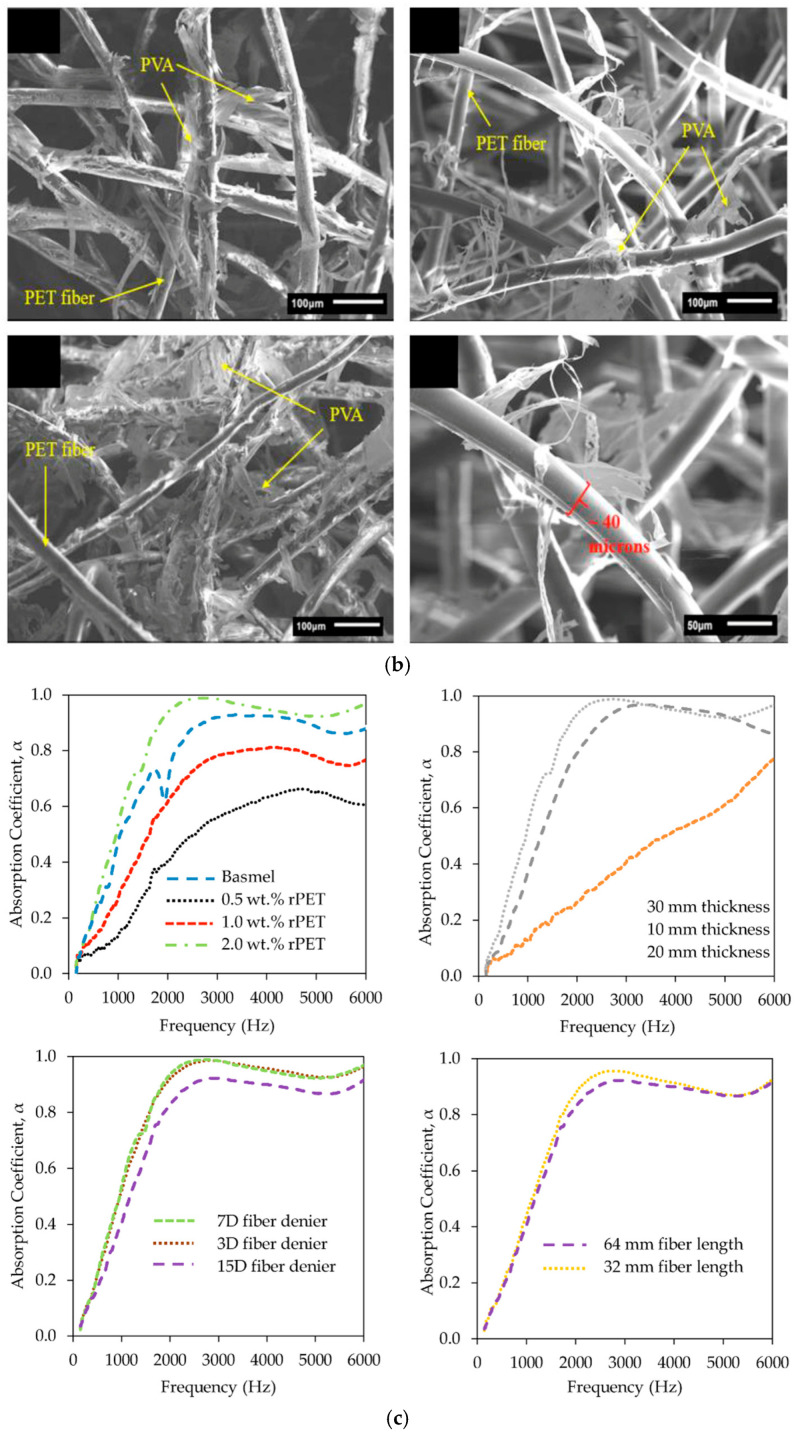
Recycled rPET aerogels for acoustic applications: (**a**) polymer recycling and fabrication process; (**b**) SEM microstructures at 0.5, 1.0 and 2.0 wt.% fiber concentration (7D, 64 mm), reproduced with permission from [18], MDPI, 2018; fiber surface morphology and (**c**) sound absorption as a function of fiber concentration, thickness, density and length, compared with commercial foam absorbers, reproduced with permission from [18], MDPI, 2018.

Recycled polymers such as PET [151], PU foams [152] and other post-manufacturing plastic materials are increasingly used in sound-absorbing applications as sustainable solutions. The polymer waste material can be recycled via mechanical and chemical processes to produce porous acoustic materials [133,153], panels or composites which perform at similar levels to traditional synthetic acoustic absorbers in terms of acoustic properties. This is demonstrated schematically in Figure 10a.

Recent studies on recycled polymer aerogels further demonstrate this potential. For example, Hong Wei Koh et al. [18] developed (rPET) aerogels from plastic waste for sound and thermal insulation. SEM images (Figure 10b) show that rPET aerogels exhibit a 3D interconnected macroporous network (>50 nm) with uniform poly(vinyl alcohol) (PVA) distribution. Increasing fiber concentration from 0.5 to 2.0 wt.% induces structural densification, reducing pore size while increasing tortuosity and airflow resistance. This microstructural shift directly enhances thermoviscous dissipation which governs sound attenuation [51,54]. The absorption spectra in Figure 10c confirm the structural dependence of sound absorption performance. The 2.0 wt.% rPET aerogel exhibits 20% to 30% higher absorption than the 1.0 wt.% sample, reaching an NRC of 0.45. Increased thickness shifts absorption toward lower frequencies by extending the propagation path while reduced fiber denier enhances performance due to higher specific surface area [26,154]. Shorter fibers slightly improve absorption through increased entanglement and internal friction. These results demonstrate that sound absorption efficiency in recycled polymer aerogels is controlled by microstructural engineering rather than polymer chemistry alone.

The use of recycled polymers presents an option for valorizing waste materials in acoustics, but in order to maximize their performance, proper management of the degree of porosity, fiber structure [42], density and thickness must be observed [26,134]. Too much densification or very fine fibers can decrease the connectivity of the pores, thus preventing airflow movement. From a polymer-specific sustainability standpoint, the environmental value of recycled polymer absorbers depends not only on waste origin but also on processing intensity, binder integration (e.g., PVA) and end-of-life recyclability [18,148,155].

Aerogel production and fiber reconstitution may bring additional energy consumption, potentially offsetting circularity savings [18,156]. Consequently, future recycled polymer acoustic systems must favor low-energy processing pathways, limited binder use and closed-loop recyclability to ensure that acoustic performance matches with genuine circular polymer engineering [157,158,159].

### 4.3. Recycled Rubber and Composite Systems

**Figure 11:**
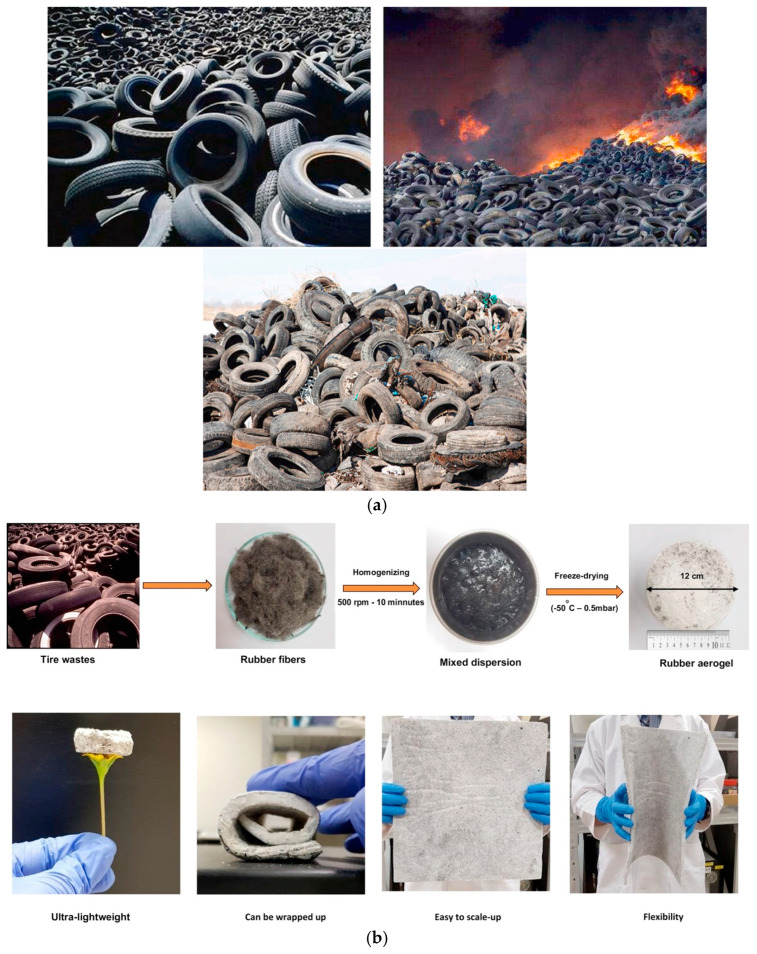
(**a**) Accumulation of end-of-life tires in open dumping sites, reproduced with permission from [160], Elsevier, 2024, and (**b**) fabrication route of rubber aerogels, reproduced with permission from [161], Elsevier, 2020.

End-of-life tires represent a persistent environmental burden due to their accumulation, fire risk and potential release of hazardous substances [160]. The impact illustrated in Figure 11a emphasizes the necessity of converting tire waste into functional, high-value materials rather than low-grade fillers or fuel substitutes.

Recycled rubber has emerged as a promising candidate for acoustic applications due to its intrinsic viscoelastic damping, mechanical resilience and large-scale availability [131,162,163]. Several researchers, including Quoc Ba Thai et al. [161], have explored the conversion of waste tire fibers into lightweight porous aerogels with promising thermal insulation and sound absorption properties. The freeze-drying process preserves continuous porous architecture without requiring complex supercritical treatment, enabling structural stability with reduced processing complexity (as shown in Figure 11b). The resulting materials combine low bulk density with mechanical flexibility [134,162], making them suitable for building-scale insulation systems.

Rubber-based absorbers typically exhibit effective low- to mid-frequency sound absorption, attributed to their high density and internal damping mechanisms [161]. To facilitate an equitable comparison of the acoustic performance of rubber materials with other sound-absorbing materials of varying thicknesses, areal density (ρareal = ρbulk × thickness) is used to normalize thickness effects. As shown in Table 3 the NRC value rises for both rubber fiber content and material thickness.

This is due to the increase in rubber areal density and the improvement of the interlinked porous network of the rubber material stretch. Increased rubber fiber fraction leads to higher internal friction and improved viscoelastic damping [164,165], which promotes the viscous and thermal dissipation of sound energy [52,53]. Also, greater thickness increases the sound dissipation path in the porous structure, allowing for more interaction of the sound waves with pore walls and therefore improves viscoelastic sound absorption. Compared to their low bulk density, rubber-based materials have competitive sound absorption which explains why the sample with 30 mm thickness (RAS12) has the highest NRC value of 0.56.

However, translating laboratory results into actual implementation involves overcoming some unique challenges related to the properties of each material. Scrap tires have a variety of chemicals, carbon black, sulfur links and process chemicals that can cause variations in their mechanical properties and acoustic response [131,134,166,167]. Even after optimization, the greater density of rubber compared to minerals or plants could lead to greater load bearing especially in the case of extensive use [18,161,168]. The inclusion of binders like PVA represents another challenge as well. Key is the lack of quantified information regarding the durability of rubber materials exposed to humidity, ultraviolet rays and temperature changes [161,169,170]. Additionally, the sustainability of recycled rubber cannot be claimed merely based on initial NRC ratings but also in terms of durability, safety from emissions and practical applications towards the end of life. Improvement in rubber sound absorber panels depends not only on proper purification of raw materials but also on their emissions and life cycle assessments. To provide a structured comparison of the recycled material systems discussed above, Table 4 summarizes their governing mechanisms, acoustic behavior, processing requirements and sustainability constraints.

The reported low- to mid-frequency tendency reflects the behavior of recycled rubber systems most commonly investigated in the literature, rather than a general characteristic of all rubber-based sound-absorbing materials. The performance of recycled rubber absorbers remains strongly dependent on structural configuration and further comparative studies under harmonized testing conditions are required before broader generalizations can be established.

## 5. Geopolymer-Based Sound-Absorbing Materials

The use of geopolymer materials has become relatively recent but they are a promising class of materials for acoustic purposes [29,30], representing a more environmentally friendly option than traditional Portland cement binders and polymeric binders [28,29,181]. Their manufacturing process is based on the alkaline activation reaction of precursors such as aluminosilicates like fly ash, metakaolin or ground granulated blast furnace slag [28,29,30]. Geopolymers feature an amorphous 3D network, which makes it possible to create geopolymer materials with regulated porosity and mechanical properties [182,183,184]. It is worth noting that their manufacturing results in less carbon dioxide emission.

**Figure 12:**
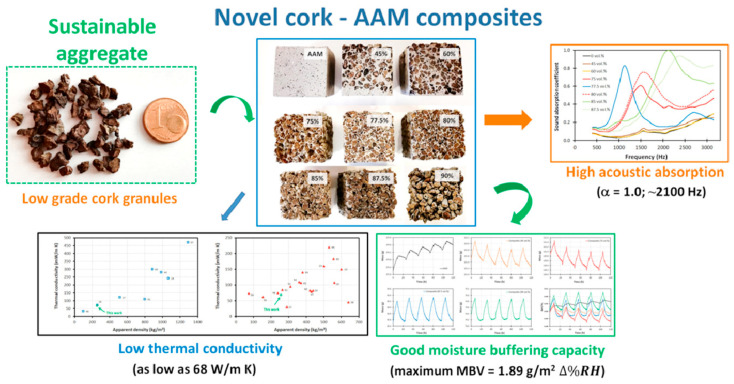
Multifunctional performance of cork–alkali-activated composites: density, thermal conductivity and sound absorption; reproduced with permission from [185], Elsevier, 2020.

The sound-absorbing capability of geopolymer composite materials is mainly determined by the pore structure in terms of porosity, pore-size distribution and connectivity [182,183,185]. In contrast to fiber-based composite systems, geopolymer composite absorbers rely on solid porous structures that dissipate thermoviscous energy through interconnected pore networks [101,185,186]. The incorporation of lightweight bio-based aggregates into alkali-activated composites enables the development of multifunctional materials with enhanced performance [184,185,187]. As illustrated in Figure 12, increasing cork content promotes a highly porous structure, resulting in improved acoustic absorption.

In this work [185], the composite containing 85 vol.% cork reaches nearly complete sound absorption (α ≈ 1.0) at approximately 2100 Hz, representing a very narrow peak of maximum absorption. In contrast, the composite containing 87.5 vol.% cork does not achieve unity absorption but exhibits a broader high-performance region, with the sound absorption coefficient remaining above 0.8 over a wide frequency range, approximately from 2265 Hz to about 3150 Hz, which corresponds to the upper limit of the tested range. This indicates that, while the 85 vol.% composite achieves a sharper and more pronounced maximum, the 87.5 vol.% composite provides more stable and broadband high absorption performance across the high-frequency region.

The geopolymer composites exhibit very low apparent densities (around to 168 kg/m^3^) and low thermal conductivities (about to 68 mW/m·K), placing them among the most lightweight and thermally efficient systems reported. These porous composites also show good moisture buffering, helping regulate indoor humidity. This multifunctional behavior improves energy efficiency, sound absorption performance and indoor comfort, supporting more sustainable building materials [29,183,185]. Beyond cork incorporation, a wider range of lightweight and waste-derived fillers has been explored to enhance the sound absorption performance of geopolymer systems [187,188]. These additives increase porosity and pore connectivity, improving airflow resistivity and sound absorption, while acting as pore-forming agents for better microstructural control [27,181,184].

Yet, one of the main problems in geopolymer absorbers is the compromise between porosity and mechanical strength [182,183]. Increased porosity increases sound absorption, especially at mid-to-high frequencies, but decreases the mechanical strength [182,183]. They are interrelated and need to be well-controlled in terms of the ratio between liquid and solid phases, curing conditions and foaming procedure [182,189].

The most frequently used foaming technique uses hydrogen peroxide or aluminum powder. Yet, hydrogen peroxide requires careful handling due to its oxidizing properties, while aluminum powder may present dust-related safety concerns. Nevertheless, sound absorption behavior does not rely only on material features.

The comparatively limited available literature on geopolymer sound absorption materials partly reflects the fact that most geopolymer research has historically focused on mechanical, thermal and durability performance, while dedicated acoustic investigations remain relatively scarce.

## 6. Additively Manufactured Sound-Absorbing Materials

AM technology has proved to be an excellent tool for developing sophisticated sound-absorbing materials based on the ability to manipulate the geometric parameters inside the structure responsible for sound dampening [36,190]. As opposed to regular porous materials in which sound absorption relies heavily on material properties [32,191], with AM technology, geometry becomes a primary variable to overall absorption behavior. Controllable structural parameters include pore size, connectivity and strut thickness [36,192,193]. Controlling such features makes it possible to tune airflow resistivity and sound energy losses in AM-created lattices to obtain targeted sound absorption at specific frequencies [173], which cannot be achieved using traditional fibers or foam materials.

Regarding sustainable aspects, one must note that AM technology allows manufacturers to reduce the volume of material waste and to work with eco-friendly materials, including natural polymers such as polylactic acid (PLA) and recycled thermoplastics [173,194,195]. Yet, the environmental impact of AM depends greatly on material choice and power usage. Using AM technology to work with sustainable materials may prove to be an effective method to develop environmentally friendly acoustic systems [195]. There have been multiple recent studies regarding AM-produced acoustic materials, including lattice materials, micro-perforated plates and even acoustic metamaterials with improved thermoviscous energy losses [196].

According to Li et al. [197], therefore, they enable the production of customized sound-absorbing structures. Recent developments in additively manufactured acoustic materials have expanded beyond conventional porous structures to include lattice and TPMS-based architectures whose sound absorption properties can be optimized through careful control of geometric parameters and structural topology.

Multi-material AM enables the integration of materials with different mechanical and acoustic properties within a single structure, expanding the design possibilities for sound-absorbing systems.

### 6.1. Additive Manufacturing Techniques and Material Compatibility

**Figure 13:**
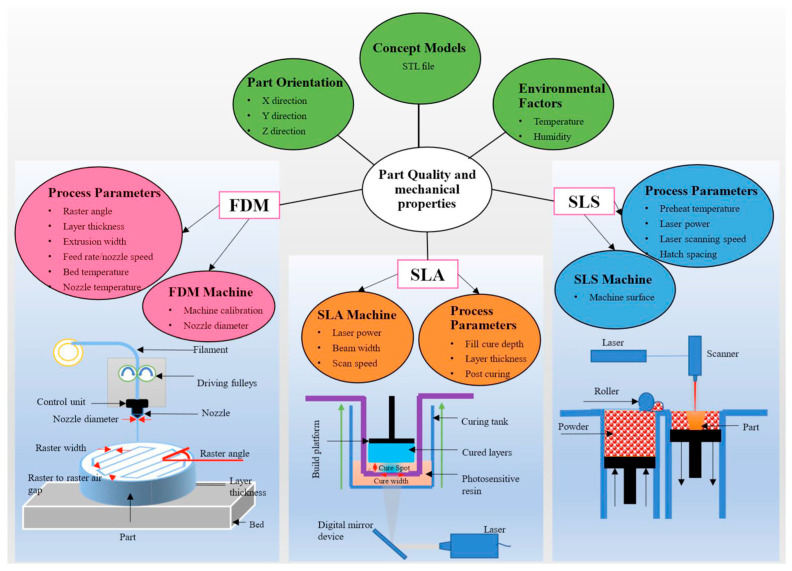
Schematic comparison of key process parameters for three additive manufacturing techniques: FDM, SLA and SLS; reproduced with permission from [198], MDPI, 2021.

Among the various AM techniques, fused deposition modeling (FDM), stereolithography (SLA) and selective laser sintering (SLS) are the most widely used for acoustic applications due to their complementary capabilities. Their typical processes are illustrated in Figure 13.

FDM is widely used due to its low cost, broad material availability and simple processing. Thermoplastic filaments such as PLA [199], acrylonitrile–butadiene–styrene (ABS) [171] and increasingly recycled or bio-based polymers are printed into lattices [200], honeycombs or graded infill patterns [194,201], providing controllable airflow resistivity [47,91]. Limitations include nozzle-dependent resolution and surface roughness, which may reduce high-frequency absorption [47,171,202]. In turn, SLA is based on the selective photopolymerization of liquid resins using ultraviolet light [203]. Compared to FDM, SLA provides higher resolution and smoother surface finishes, enabling the fabrication of micro-perforated panels, fine lattice structures and resonant cavities with precise dimensional control [32,198,203]. These characteristics make SLA particularly attractive for frequency-selective absorbers and acoustic metamaterial designs. Finally, SLS is a powder-based AM technique in which a laser selectively fuses polymer particles to form solid structures [198,204]. High equipment costs and energy consumption, however, limit its large-scale application.

AM enables the fabrication of complex, self-supporting geometries without auxiliary support, making it particularly suitable for producing 3D porous networks and intricate lattice architectures [32,198,205]. AM methods for sound absorption panels present considerable design adaptability, yet they are constrained by several factors [32,38,206]. FDM is characterized by reduced printing resolution and surface roughness, which adversely affects sound absorption performance at high frequencies [207,208]. Conversely, SLA provides high precision; however, it requires photopolymer resins, thereby raising questions regarding both longevity and environmental impact [196,198]. SLS facilitates the creation of lattice structures optimized for sound absorption, although its application is restricted by substantial equipment expenses and energy requirements [198,204,206,209]. Across all AM techniques, sound absorption performance is sensitive to processing parameters such as layer thickness and infill strategy [38,196,206]. These factors influence geometric accuracy and contribute to variability in sound absorption response, highlighting the need for improved process control and standardization [31,195,196].

### 6.2. Architected Geometries for Sound Absorption

**Figure 14:**
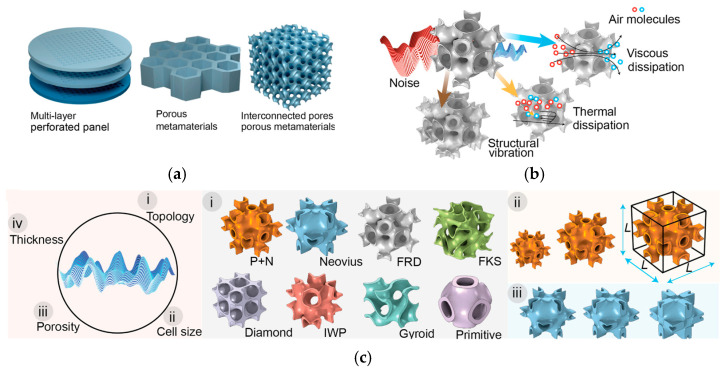

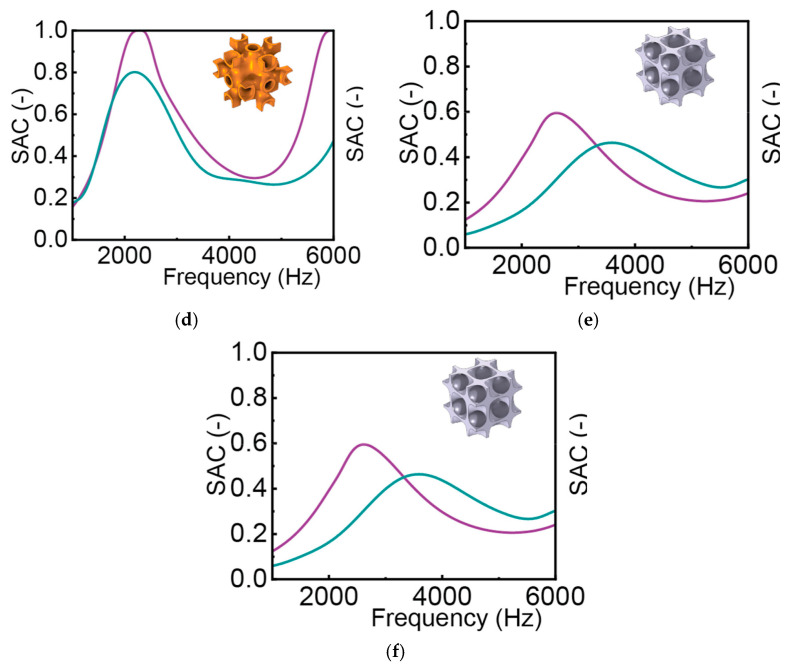
Evolution and performance of advanced porous metamaterial absorbers: (**a**) structural transition toward interconnected architectures, (**b**) multiscale dissipation mechanisms, (**c**) key design parameters of TPMS lattices and (**d**–**f**) experimental validation of numerical absorption models, reproduced with permission from [210], Elsevier, 2025.

The creation of various sound absorption structures, such as slotted patterns, perforated panels and architected cellular materials, has been made possible by recent developments in AM [43]. Figure 14a illustrates architected cellular materials, also known as lattice metamaterials, which have attracted significant attention due to their multifunctional characteristics and tunable sound absorption performance.

Several successful examples of hybrid acoustic absorbers have been reported in the literature. These include lattice structures filled with polyurethane foam [37,211,212], micro-perforated panels coupled with porous backing layers and TPMS architectures combined with viscoelastic materials [210,213,214]. In these systems, the architected geometry promotes viscous and thermal losses while the secondary material contributes additional damping and broadband absorption [53,215]. Such synergistic effects often result in higher sound absorption coefficients and wider effective frequency ranges than those achievable with single-material designs.

Unlike traditional fibrous or foam-based absorbers, AM acoustic materials depend primarily on their internal architecture to manage sound attenuation [32,216]. Within these materials, sound absorption is mainly regulated by geometrical considerations rather than the physical properties of the building material, hence the transition to geometry-oriented acoustic design [43,215]. The fundamental geometrical variables, such as unit cell structure [217], connectivity of pores [35], channel diameter and cavity dimension [218,219], regulate the flow resistivity and the surface impedance.

Moreover, other geometrical features, such as tortuosity and porosity gradients, broaden the scope of design and offer enhanced sound absorption performance adjustment. Thermoviscous dissipation constitutes the dominant attenuation mechanism in such architected systems [210]. Sound energy is dissipated through viscous friction and thermal exchanges within confined air volumes embedded in complex geometries (as shown in Figure 14b).

Structural vibration of the solid framework generally plays a secondary role, unless intentionally designed as part of a coupled resonant mechanism [101,210]. The geometry-driven acoustics of these structures are illustrated in Figure 14c. Experimental evidence indicates enhanced absorption in the mid- to high-frequency range (approximately 2–6 kHz), whereas reliable low-frequency performance below 500 Hz remains limited. In general, effective low-frequency absorption requires additional design strategies, including resonant cavities, graded porosity, multi-scale architectures, or hybrid metamaterial configurations [210].

Experiments and simulations indicate that the location and intensity of absorption peaks depend significantly on cavity dimensions [220], channel connectivity [219], hydraulic diameter and tortuosity [24,38,221]. The findings show that geometry-based tuning of the sound absorption properties is possible regardless of the chemical composition of materials used. Nevertheless, the following disadvantages also arise as shown in Figure 14d,f. The low-frequency absorption is limited by the disparity of wavelength in relation to the characteristically small dimensions of the lattice [101]. Although an increase in the tortuosity or decrease in the size of the channels results in higher thermoviscous dissipation, excessively high airflow resistivity may interfere with wave propagation [101,210]. It is also notable that existing research is based almost exclusively on impedance tube measurements and computer simulations. Although these tests provide valuable insight into intrinsic acoustic behavior, they may not accurately represent diffuse-field environments encountered in buildings and transportation systems. Factors such as incidence-angle distribution, panel dimensions, mounting configuration, edge effects and room acoustics can significantly influence practical performance. Therefore, broader validation through reverberation chamber testing and full-scale field studies remains necessary. Additionally, full-scale experimental confirmation at the architectural level is still rare [222,223], which leaves certain doubts concerning the constructability, physical durability and efficiency of acoustic treatment with AM [222,224].

### 6.3. Hybrid Geopolymer-Based Architectures and Future Perspectives for Low-Frequency Sound Absorption

Even though much has been gained in terms of effectiveness by developing natural and recycled acoustic sound absorbers, the latter’s efficiency is mostly observed at medium and high frequencies. As was mentioned above, the major principles for sound dissipation in such materials are viscosity and thermal losses, which take place in porous media.

Nevertheless, low-frequency sound waves have very long wavelengths compared to other waves; therefore, they cannot interact effectively with conventional porous materials [101,225]. That is why absorbing sound waves at such frequencies often calls for considerable thickness of materials which in turn increases their cost and weight [26,127]. In addition, many natural sound absorbers suffer from moisture sensitivity and lack of fire resistance.

As a possible way to overcome these drawbacks, hybrid structures based on geopolymeric matrices combined with bio-based or recycled fillers have appeared recently [226]. Firstly, geopolymers possess some considerable advantages compared with purely organic sound-absorbing materials, including good strength and high durability under harsh environmental conditions [184,227].

However, the addition of lightweight bio-fillers, e.g., cork, natural fibers, agriculture waste, or recycled material allows the introduction of extra porosity into the material structure, providing increased thermoviscous sound absorption [187,188,226]. In other words, new materials can provide a good combination of sustainability due to the use of renewable or recycled fillers and high durability associated with geopolymeric matrices.

A further potential benefit offered by using AM techniques lies in its application of sustainable acoustic materials [36]. While in conventional porous absorbents, acoustic properties depend mainly on material composition, AM makes it possible to control such geometric factors as pore size, connectivity, channel diameter, tortuousness, cavity size and distribution of graded porosity [219,228].

The use of AM becomes especially important for the low-frequency range. For the purpose of effective acoustic absorption in the low-frequency range, porous absorbents need considerable thickness. In turn, using AM, one can create resonators like Helmholtz-resonator-like cavities [55,57], micro-perforated plates [70,214], graded lattices [196,205], triply periodic minimal surfaces (TPMS) and metamaterials [222,228]. With the help of specially engineered geometry, new resonances and impedance matching are generated which contribute greatly to enhancing absorption in the range of frequencies that are inaccessible to conventional porous materials. Therefore, significant absorption in the low-frequency range is possible without increasing material thickness excessively [225,229].

Additionally, it is possible to fabricate hierarchical and multiscale architectural designs whereby various features work together on different frequency bands [6,46]. This means that there will be both broadband absorption capabilities and low frequency absorption, thus improving overall performance while reducing material needs.

Finally, the layer-by-layer construction method allows for combining various materials within a unitary element, making it possible for the creation of hybrid acoustic designs involving geopolymer materials, recycled plastic and bio-based materials.

For these reasons, the development of hybrid geopolymers combined with AM techniques could represent an attractive way forward when designing future sustainable acoustic materials [230,231]. In addition to the benefits offered by geopolymers, such as stability and fire-resistant characteristics, bio-based and recycled materials provide sustainable solutions as well as porous structures. AM techniques add value by virtue of their capabilities in creating resonant structures at specific frequencies [32,192,228]. Future research is likely to involve the development of multifunctional materials, combining acoustic performance, strength and fire resistance with environmental sustainability and other desirable characteristics.

## 7. Comparative Analysis of Conventional and Sustainable Sound-Absorbing Materials

Traditional porous sound absorbers such as mineral wool [142], glass fiber [232] and polymer foams continue to set industry standards due to their cost-effectiveness [233,234], scalability and proven broadband sound absorption behavior based on viscous–thermal losses in fiber-based or open-cell media [24,41]. Density, thickness and airflow resistivity are key design variables of traditional sound absorbers [26,127,134]. Conversely, natural and recycled materials aim to reduce environmental impact while maintaining comparable sound absorption efficacy, particularly in the higher frequency range [16,20]. Although these materials align with circular economy principles, their broader implementation is still impeded by variations in raw material properties [120,235], challenges related to moisture resistance, stability and durability, alongside difficulties concerning flame retardancy [149,159,206].

Geopolymer-based absorbers and those fabricated through AM have recently emerged as promising inorganic alternatives due to their controlled porous structure, thermal stability and inherent fire resistance [29,128,182,236]. AM enables the precise design of complex pore architectures and graded internal geometries, offering enhanced control over sound absorption behavior [36,192]. Their sound absorption performance is primarily governed by pore connectivity [67,237], pore-size distribution [23,146], printing strategy and foaming approach [34,237]. However, optimizing the balance between porosity, acoustic efficiency and mechanical strength remains a major challenge. Unlike conventional porous absorbers, AM-based acoustic systems rely not only on material properties but also on geometry-driven sound attenuation. This is made possible through careful manipulation of lattice topologies and other structural features [210], which allow for frequency-specific sound absorption, particularly at lower frequencies, while maintaining reduced material thickness [101,238]. On the other hand, the widespread use of these technologies is currently hindered by high production costs [239,240], significant energy consumption [120,241], difficulties in scaling up and a lack of long-term performance validation.

To summarize, each approach involves different types of optimizations in Table 5.

## 8. Research Gaps and Future Perspectives

Despite the major advancements made in the development of sustainable sound-absorbing materials, there are technical and physical constraints that prevent their broader acceptance and implementation [155,206]. Further progress in this area is contingent upon adopting a holistic approach to developing materials that can satisfy acoustic requirements, demonstrate long-term performance, comply with environmental testing requirements and be mass-produced [9,39,121]. This means that further innovations will only come as a result of joint efforts across different scientific disciplines.

### 8.1. Standardization and Test Procedures

One limitation that has been identified consistently throughout the studies is the absence of standard preparation and test procedures. Factors such as differences in thickness, density, mounting technique and frequencies hinder meaningful comparison of results [26,93,94]. It is therefore critical that future studies consider standardized characterization techniques, which include providing measurements of the airflow resistivity [25], porosity [23], tortuosity and errors associated with each experiment [12,24].

For residue-based absorbers, future standardization efforts should incorporate batch-to-batch variability assessments through routine characterization of moisture content, particle size distribution, density, porosity, airflow resistivity and chemical composition. Such protocols are essential to ensure reproducible acoustic performance and to facilitate industrial-scale implementation. Similarly, fire performance represents a critical challenge for the commercialization and regulation of textile and bio-based acoustic materials. Therefore, compliance with relevant building fire safety standards, while maintaining acoustic performance and sustainability objectives, must be addressed.

### 8.2. Low-Frequency Sound Absorption

While numerous sound absorption materials derived from biological sources or recyclable materials have shown comparable performances at medium and high frequencies, low-frequency attenuation continues to pose a significant challenge [155,206]. This problem is primarily attributed to limitations inherent in geometrical scaling effects and the inadequacy of resonance characteristics in light porous materials [32,206]. Future innovations in this field will likely result from innovative approaches involving the combination of porous dissipation properties and resonance phenomena [55,244]. Potential developments in this regard could include embedding Helmholtz cavities, using graded porosity designs and implementing AM resonance-based cores in bio-sourced absorptive matrices [128,191].

**Figure 15:**
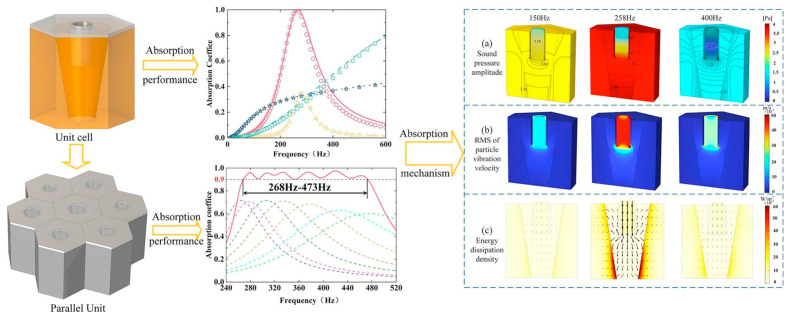
Low-frequency broadband sound absorption performance and mechanism of gradient-perforated porous acoustic metamaterials with embedded neck resonators, reproduced with permission from [238], Elsevier, 2025.

Recent studies suggest that gradient perforation in porous metamaterials, when combined with embedded resonator necks, can enable efficient broadband low-frequency absorption while maintaining compact material thickness [238], as shown in Figure 15.

Although gradient-perforated metamaterials and embedded resonator architectures show considerable promise for low-frequency sound absorption, their practical implementation remains uncertain. Many proposed designs rely on complex geometries that may require advanced manufacturing processes, increasing production costs, energy consumption and quality-control requirements. Consequently, future studies should evaluate not only acoustic performance but also manufacturability, scalability, durability and techno-economic feasibility.

### 8.3. Durability, Fire Safety and Environmental Stability

An important limitation for the use of bio-based sound absorption materials is the requirement of environmental stability. The susceptibility to moisture [104,105], degradation [176,253], exposure to cycling humidity and poor inherent flame retardancy affect meeting building code standards [105,126,150]. While chemicals might improve fire and moisture resistance [105,150,254,255], they might adversely impact the degradation rate or pose environmental/toxicological problems [256]. In future studies, efforts should be made towards the design of bio-based flame retarding agents, eco-friendly surface treatments and accelerated aging tests which consider mechanical strength and acoustic properties at the same time [32,113,206,257]. Data on long-term stability needs to be developed.

### 8.4. Life Cycle Assessment (LCA) and Circularity Metrics

Beyond sound absorption performance and durability, environmental validation remains a critical requirement for sustainable acoustic materials. Many studies classify materials as “eco-friendly” based primarily on renewable or recycled origin [82,255], without rigorous quantification of embodied energy [129,258], carbon emissions [39,259], manufacturing impacts or end-of-life scenarios [260,261]. Such qualitative claims limit objective comparison with conventional absorbers and may hinder regulatory approval.

**Figure 16:**
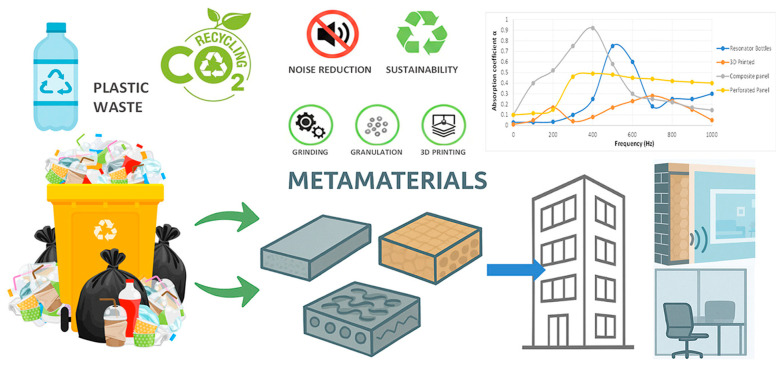
Circular pathway for converting recycled PET bottles into acoustic metamaterials, showing processing, design, acoustic testing and building-scale integration, reproduced with permission from [177], Elsevier, 2026.

Recent developments in rPET-based sound absorption metamaterials demonstrate how circular design principles can be integrated with sound absorption optimization [130,260]. By converting plastic bottle waste into architected resonant structures for building noise control, these systems establish a closed-loop valorization pathway linking waste recovery, material processing, structural design and performance evaluation (Figure 16). However, additional processing steps, AM energy demand [113,262], feedstock variability [263] and fire safety compliance introduce important sustainability trade-offs that require systematic assessment [125,126].

Although numerous studies describe environmental advantages associated with renewable or recycled acoustic materials, comprehensive cradle-to-grave LCAs directly comparing bio-based, recycled, geopolymer or additively manufactured sound absorption systems remain limited. As a result, current sustainability claims are often based on material origin or waste valorization potential rather than fully quantified life cycle impacts.

### 8.5. Digital Design and Multi-Physics Optimization

AM technology allows a high degree of geometric freedom in producing sound absorption materials [43,128]. However, much of the research relies on empirical parameter tuning rather than comprehensive optimization methods [25,54,246]. Future advancements are expected to favor multi-objective and multi-physical optimization frameworks which will enable the simultaneous optimization of acoustic characteristics, mechanical behavior, thermo-stability and material efficiency. This is crucial for real-world applications, as sound absorption materials must withstand specific mechanical and thermal loads. Advanced computational methods, including machine learning and artificial intelligence, are becoming essential for achieving optimal acoustic performance [210,264]. Additionally, integrating digital design with sustainable or bio-sourced materials is pivotal for developing environmentally friendly acoustic materials.

### 8.6. Hybrid and Multi-Material Systems

**Figure 17:**
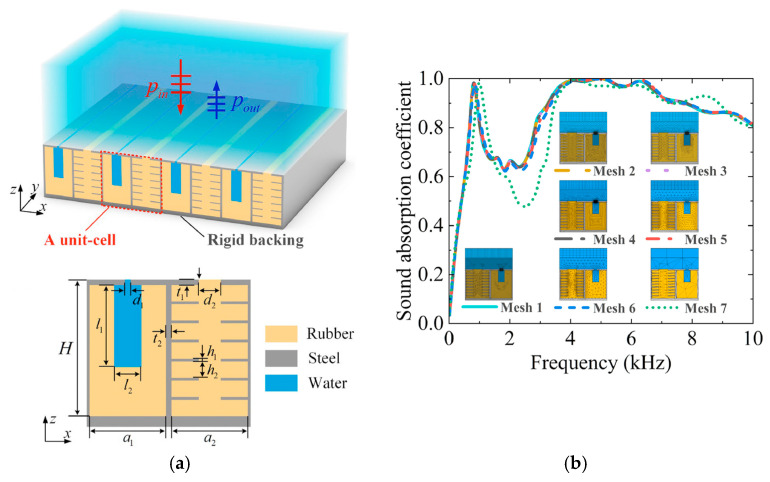
(**a**) A schematic diagram of the proposed hybrid metamaterial including the cross-section of a representative unit cell in the plane, and (**b**) mesh independence verification: variation in sound absorption coefficients with mesh density, reproduced with permission from [266], Elsevier, 2025.

None of the existing materials provides the combination of broadband absorption, high mechanical strength, resistance to fire and ecological sustainability. Hybrid structures with several functional components have been proposed as a solution to create next-generation sound absorption structures [68]. A combination of different structural materials could result in the creation of multifunctional absorbers including bio-fibers and recycled plastics, viscoelastic rubber dampers or resonant structures created by AM and placed inside porous media [68,231,265]. Such a design concept can be exemplified by a hybrid sound absorption metamaterial based on the combination of a rubber Helmholtz resonator and rubber plates, as described in Figure 17a. The unit cell geometry is presented in the figure. A typical example of how the hybrid structure is formed is demonstrated in Figure 17b when the local resonances and structural vibrations interact. It can be seen numerically that multiple absorption peaks are provided in such a system for broad frequency spectra [266]. These results show that the development of future acoustic technology should be focused not on the replacement of conventional materials but on the design of hybrid structures.

## 9. Conclusions

Sustainable sound-absorbing materials have emerged as promising alternatives to conventional sound absorbers, driven by increasing environmental concerns and circular economy objectives. Bio-based and recycled materials offer significant environmental benefits while achieving sound absorption performance comparable to traditional absorbers, particularly at medium and high frequencies. Geopolymer-based materials provide additional advantages, including enhanced thermal stability, fire resistance and mechanical robustness. Meanwhile, AM enables the development of architected acoustic structures with tailored geometries and frequency-selective absorption characteristics. Despite these advances, several challenges continue to limit large-scale implementation, including insufficient low-frequency absorption, durability concerns, moisture sensitivity, fire resistance requirements, raw material variability and the lack of comprehensive LCAs. Furthermore, balancing sound absorption performance, mechanical integrity and environmental sustainability remains a critical challenge across all material classes. Future research should focus on the development of hybrid acoustic systems that combine sustainable porous materials, geopolymer matrices and geometry-engineered architectures. The integration of advanced manufacturing technologies, multiscale characterization and multi-physics optimization will be essential for achieving durable, high-performance and environmentally responsible sound-absorbing solutions.

## Figures and Tables

**Table 1:** Comparative acoustic performance and governing parameters of natural fiber-based absorbers (adapted from Berardi et al.) [11].

Materials	Thickness Range (m)	Low-Frequency Performance (125–250 Hz)	Mid–High Frequency Performance (500–2000 Hz)	Maximum NRC	Dominant Governing Parameters	Key Observations
Kenaf	0.04–0.06	Low (<0.30)	Very high (up to 0.95 at 1000–2000 Hz)	0.70	Thickness, density, fiber packing	Strong thickness dependence: dense panels enhance mid-frequency absorption
Wood fibers	0.03–0.06	Moderate to low	High (>0.90 at 2000 Hz)	0.60	Fiber morphology, mineralization	Mineralized wood shows reduced porosity and lower absorption
Hemp	0.03	Very low	Moderate (≤0.70 at 2000 Hz)	0.40	Fiber diameter, airflow resistivity	Thin panels are insufficient for broadband absorption
Coconut fiber	0.05–0.10	Moderate (improves with thickness)	High (around 0.94 at 2000 Hz)	0.75	Thickness, inter-fiber voids	0.10 m panels show strong broadband improvement
Cork	0.03	Very low	Moderate at high frequency	0.30	Cellular closed structure	Limited performance due to lower interconnected porosity
Cane	0.04–0.08	Low to moderate	Moderate to high (≤0.89)	0.60	Bark vs. wood fraction	Bark-rich panels show better absorption
Cardboard	0.10	Moderate	Moderate to high	0.50	Layered structure	Performance linked to interlayer air gaps
Sheep wool	0.04–0.06	Moderate	Very high (around 0.95)	0.70	Fiber crimp, tortuosity	Excellent mid-frequency dissipation

**Table 2:** Comparison of structural features and performance of bio-based acoustic materials.

Parameters	Natural Fibers	Agricultural Residues	Mycelium Composites
Structural Formation	Mechanical packing of fibrous networks [11].	Compacted particles with or without bio-binders [96,115].	Biologically grown hyphal matrix binding substrates [110,111].
Primary Governing Mechanism	Porosity [23,116], fiber tortuosity [24], airflow resistivity [25].	Inter-particle voids, particle size distribution [23,100], airflow resistivity [25].	Hyphal network connectivity [112,117], multiscale porosity [24], airflow resistivity [25].
Density Range (kg/m^3^)	(30–200) [11]	(80–250) [95]	(120–170) [112,118]
Mid–High Frequency Absorption	High (often α > 0.7 with optimized thickness) [11,51].	Moderate to high [97,100].	High (α up to 0.8–0.9 under optimized growth) [109,119].
Low-Frequency Performance	Limited; thickness-dependent [11,120].	Limited; compaction dependent [96,121].	Limited; thickness-dependent [109,114].
Microstructural Tunability	Controlled via density and fiber orientation [11,94].	Controlled via particle size and binder content [96,97].	Tunable via fungal strain, substrate type, incubation time [109,110].
Mechanical Stability	Moderate; improves with densification [51,122].	Variable; trade-off with porosity [23,95,96].	Moderate; strain and substrate dependent [112,119].
Moisture Sensitivity	High (hygroscopic fibers) [123].	High (lignocellulosic particles) [121,123].	Moderate to high; requires stabilization [106,119].
Binder Requirement	Often required [10,124].	Frequently required [96,97].	Natural biological binder (mycelium) [111,119].
Main Limitations	Low-frequency inefficiency [10,11]; fire resistance [125,126].	Durability porosity trade-off [84].	Durability [114,118]; scalability [41].
Key Optimization Strategy	Thickness and airflow resistivity control [25,127].	Particle engineering [121,128].	Growth parameter optimization [8,112].

**Table 3:** Acoustic properties of recycled rubber materials with different rubber fiber contents and thicknesses. Sample notation (RAS03–RAS12) adapted from [161].

Samples	Rubber Fibers (wt.%)	PVA (wt.%)	Density (mg·cm^−3^)	Thickness (mm)	NRC	Areal Density (g·m^−2^)
RAS03	3.0	–	57	15	0.35	855
RAS04	4.0	–	75	15	0.38	1125
RAS05	5.0	1.0	91	15	0.42	1365
RAS06	6.0	–	108	15	0.45	1620
RAS07	7.0	–	126	15	0.48	1890
RAS08	5.0	1.0	91	10	0.31	910
RAS09	5.0	1.0	91	15	0.42	1365
RAS10	5.0	1.0	91	20	0.49	1820
RAS11	5.0	1.0	91	25	0.52	2275
RAS12	5.0	1.0	91	30	0.56	2730

**Table 4:** Comparative assessment of recycled material systems for sound absorption.

Categories	Governing Mechanism	Frequency Performance	Structural Control	Processing Complexity	Key Limitations	Sustainability Considerations
Recycled Textiles	Airflow resistivity and fiber entanglement [93,137].	Mid–high frequency dominant [145,149].	Density and compaction tunable [16,93].	Low–moderate (mechanical processing) [138,149].	Fire resistance [125], aging [150].	Low embodied energy [149]; scalability favorable [93,145]; durability underexplored [138].
Recycled Polymers (rPET, PU)	Thermoviscous dissipation via engineered porosity [8,133].	Broadband (structure-dependent) [171,172,173].	High microstructural tunability (denier, thickness, concentration) [133,148,174].	Moderate–high (aerogel/fiber reconstitution) [18,173,175].	Binder effects and pore connectivity control [23,172,176].	Circular potential depends on processing energy and recyclability [129,177].
Recycled Rubber Aerogels	Mass-related damping and viscoelastic losses [134,178].	Low–mid frequency dominant [19,20,134].	Controlled mainly via areal density [19,166].	Moderate (freeze-drying) [166,179].	High intrinsic density and compositional heterogeneity [20,178,180].	Durability and aging data limited [134,167,170].

**Table 5:** Comparative analysis of conventional, bio-based/recycled, AM and geopolymer-based acoustic materials.

Aspects	Conventional Porous Materials	Bio-Based/Recycled Materials	AM and Geopolymer-Based Acoustic Materials
Design principle	Material-property-driven porous dissipation [54,242].	Sustainable substitution of porous systems [16,20,134].	Geometry-driven and pore-engineered acoustic systems based on AM architectures and porous geopolymer matrices [169,182,210].
Primary mechanism	Viscous–thermal losses in fibrous and open-cell networks [23,52,53].	Similar porous dissipation mechanisms [54,243].	Thermoviscous dissipation through engineered pore networks [210,237], tortuous channels and resonant geometries [24,219,244].
Key parameters	Density [94], thickness [26,51], airflow resistivity [25,51], fiber diameter [51,106].	Porosity control [51,116], fiber morphology [42,245], raw material consistency [158,200].	Pore architecture [191], pore connectivity [147,237], tortuosity [24,221], printing parameters [38,189,246], porosity and mix design [84,191,247].
Low-frequency performance	Requires increased thickness [24,79].	Similar limitation to conventional [24,242].	Tunable through lattice geometry [205,210,247], resonant cavities [55,244], foaming agents and graded porosity [183,191].
Environmental profile	Non-renewable, energy-intensive production [113,120].	Renewable/recycled [248,249] and lower embodied carbon [84,250].	Potentially low-carbon when industrial waste precursors and recycled feedstocks are used [37,181,200].
Durability and fire behavior	Well-established and standardized [13,40].	Moisture-sensitive and often requires treatment [105,170].	High thermal stability, intrinsic fire resistance and good chemical durability.
Cost and scalability	Low cost [223,239]; industrially mature [100].	Moderate and scalable with processing control [145,224].	Emerging technologies with scalability and energy-consumption challenges [32,113,185].
Design flexibility	Limited geometric tunability [120,251].	Moderate (controlled porosity) [17,174].	Very high geometric tunability and frequency-selective capability [32,185,221].
Typical applications	Large-scale and cost-driven installations [41,113].	Sustainable building applications [7,115].	Acoustic metamaterials [43,190], lightweight acoustic panels [187], multifunctional building components and customized absorbers [32,185,252].

## Data Availability

No new data were created or analyzed in this study. Data sharing is not applicable to this article.

## References

[1] Hemmat W., Hesam A.M., Atifnigar H. (2023). Exploring Noise Pollution, Causes, Effects, and Mitigation Strategies: A Review Paper. Eur. J. Theor. Appl. Sci..

[2] Mehrotra A., Shukla S.P., Shukla A.K., Manar M.K., Singh S.K., Mehrotra M. (2024). A Comprehensive Review of Auditory and Non-Auditory Effects of Noise on Human Health. Noise Health.

[3] Halperin D. (2014). Environmental Noise and Sleep Disturbances: A Threat to Health?. Sleep Sci..

[4] Lim H., Utyuzhnikov S.V., Lam Y.W., Turan A. (2011). Multi-Domain Active Sound Control and Noise Shielding. J. Acoust. Soc. Am..

[5] Licitra G., Bolognese M., Chiari C., Carpita S., Fredianelli L. (2022). Noise Source Predominance Map: A New Representation for Strategic Noise Maps. Noise Mapp..

[6] Oh J.-H., Lee H.R., Umrao S., Kang Y.J., Oh I.-K. (2019). Self-Aligned and Hierarchically Porous Graphene-Polyurethane Foams for Acoustic Wave Absorption. Carbon N. Y..

[7] Asdrubali F., Schiavoni S., Horoshenkov K.V. (2012). A Review of Sustainable Materials for Acoustic Applications. Build. Acoust..

[8] Czyżów W., Bednarski M., Drzeżdżon J. (2026). Structure-Property Correlations in the Sustainable Valorization of Bio-Based Polymers: A Critical Review of Multiple Recycling Strategies. J. Environ. Chem. Eng..

[9] Zuiderveen E.A.R., Kuipers K.J.J., Caldeira C., Hanssen S.V., van der Hulst M.K., de Jonge M.M.J., Vlysidis A., van Zelm R., Sala S., Huijbregts M.A.J. (2023). The Potential of Emerging Bio-Based Products to Reduce Environmental Impacts. Nat. Commun..

[10] Yang T., Hu L., Xiong X., Petrů M., Noman M.T., Mishra R., Militký J. (2020). Sound Absorption Properties of Natural Fibers: A Review. Sustainability.

[11] Berardi U., Iannace G. (2015). Acoustic Characterization of Natural Fibers for Sound Absorption Applications. Build. Environ..

[12] Raja J. (2013). Experimental Study on Natural Fibres for Green Acoustic Absorption Materials. Am. J. Appl. Sci..

[13] Montazeri P., Bamshad O., Aghililotf M., Singh P. (2025). Mechanical and Durability-Based Life Cycle Assessment of Rice Husk Ash Containing Concrete. Case Stud. Constr. Mater..

[14] Rodríguez Neira K., Cárdenas-Ramírez J.P., Rojas-Herrera C.J., Haurie L., Lacasta A.M., Torres Ramo J., Sánchez-Ostiz A. (2024). Assessment of Elaboration and Performance of Rice Husk-Based Thermal Insulation Material for Building Applications. Buildings.

[15] Mehrzad S., Taban E., Soltani P., Samaei S.E., Khavanin A. (2022). Sugarcane Bagasse Waste Fibers as Novel Thermal Insulation and Sound-Absorbing Materials for Application in Sustainable Buildings. Build. Environ..

[16] Seddeq H.S., Aly N.M., Marwa A.A., Elshakankery M. (2013). Investigation on Sound Absorption Properties for Recycled Fibrous Materials. J. Ind. Text..

[17] Caniato M., Cozzarini L., Schmid C., Gasparella A. (2022). A Sustainable Acoustic Customization of Open Porous Materials Using Recycled Plastics. Sci. Rep..

[18] Koh H.W., Le D.K., Ng G.N., Zhang X., Phan-Thien N., Kureemun U., Duong H.M. (2018). Advanced Recycled Polyethylene Terephthalate Aerogels from Plastic Waste for Acoustic and Thermal Insulation Applications. Gels.

[19] Smirnova O.M., Menéndez Pidal de Navascués I., Mikhailevskii V.R., Kolosov O.I., Skolota N.S. (2021). Sound-Absorbing Composites with Rubber Crumb from Used Tires. Appl. Sci..

[20] Hong Z., Bo L., Guangsu H., Jia H. (2007). A Novel Composite Sound Absorber with Recycled Rubber Particles. J. Sound Vib..

[21] Rossing T.D., Rossing T.D. (2014). Springer Handbook of Acoustics.

[22] Prasetiyo I., Gunawan, Adhika D.R. (2021). Sound Absorption Performance of Nonwoven Fabrics. J. Phys. Conf. Ser..

[23] Nimmo J.R. (2013). Porosity and Pore Size Distribution. Reference Module in Earth Systems and Environmental Sciences.

[24] Sadouki M. (2018). Experimental Measurement of the Porosity and the Viscous Tortuosity of Rigid Porous Material in Low Frequency. J. Low Freq. Noise Vib. Act. Control.

[25] Hurrell A.I., Horoshenkov K.V., Pelegrinis M.T. (2018). The Accuracy of Some Models for the Airflow Resistivity of Nonwoven Materials. Appl. Acoust..

[26] Hassan N.N.M., Rus A.Z.M. (2013). Influences of Thickness and Fabric for Sound Absorption of Biopolymer Composite. Appl. Mech. Mater..

[27] Fang Y., Hu T., Qiao L., Yu F., Zhang L., Sun H., Li C. (2025). Alkali-Activated Metakaolin-Blast Furnace Slag Blends as an Alternative Inorganic Adhesive for FRP-Based Structural Rehabilitation. J. Build. Eng..

[28] Gigar F.Z., Khennane A., Liow J.-L., Tekle B.H., Li Z. (2026). From Portland Cement to Alkali-Activated System: Advances in Wood-Cement Composites for Sustainable Building Applications. Clean. Mater..

[29] Wang S., Li H., Zou S., Liu L., Bai C., Zhang G., Fang L. (2022). Experimental Study on Durability and Acoustic Absorption Performance of Biomass Geopolymer-Based Insulation Materials. Constr. Build. Mater..

[30] Poonia M.K., Boora A. (2026). Utilization of Industrial By-Products in Sustainable Geopolymer Concrete: A Comprehensive Review. Environ. Sci. Pollut. Res..

[31] Sekar V., Fouladi M.H., Namasivayam S.N., Sivanesan S. (2019). Additive Manufacturing: A Novel Method for Developing an Acoustic Panel Made of Natural Fiber-Reinforced Composites with Enhanced Mechanical and Acoustical Properties. J. Eng..

[32] Esan T.M., Kupolati W.K., Ackerman C. (2026). Advances in Additive Manufacturing for Architectural Acoustics: Design, Materials, Validation, and Implementation Challenges in Building Construction. Addit. Manuf. Lett..

[33] Mallesh S., Hwang J., Choi H., Hong D.-J., Seok C., Kwak B., Lee S.-Y., Nam Y. (2024). Advanced Acoustic Design: 3D Printed Thermoplastic Folded Core Sandwich Structures with Porous Materials and Microperforations for Enhanced Sound Absorption. Compos. Struct..

[34] Monkova K., Vasina M., Monka P.P., Kozak D., Vanca J. (2020). Effect of the Pore Shape and Size of 3D-Printed Open-Porous ABS Materials on Sound Absorption Performance. Materials.

[35] Jiang Z., Wu K., Couples G.D., Ma J. (2011). The Impact of Pore Size and Pore Connectivity on Single-Phase Fluid Flow in Porous Media. Adv. Eng. Mater..

[36] Zhou L., Miller J., Vezza J., Mayster M., Raffay M., Justice Q., Al Tamimi Z., Hansotte G., Sunkara L.D., Bernat J. (2024). Additive Manufacturing: A Comprehensive Review. Sensors.

[37] Lu Y., Xiao J., Li Y. (2024). 3D Printing Recycled Concrete Incorporating Plant Fibres: A Comprehensive Review. Constr. Build. Mater..

[38] Hamrouni A., Rebiere J.-L., El Mahi A., Beyaoui M., Haddar M. Static Study of Bio-Based Architectural Materials Made with 3D Printing Technology. Proceedings of the Fourth International Conference on Acoustics and Vibration (ICAV2022).

[39] Sen P., Debsarkar A., Kundu D. (2026). A Snippet of Environmental Footprint, Regulatory, and Policy Considerations of CO_2_ Capture Applications Employing Bio-Based Materials. Biobased Materials in Carbon Capture Applications.

[40] Silva F.A., Peled A., Zukowski B., Toledo Filho R.D. (2017). Fiber Durability. A Framework for Durability Design with Strain-Hardening Cement-Based Composites (SHCC): State-of-the-Art Report of the RILEM Technical Committee 240-FDS.

[41] Pu Y., Hao Y., Zhang W., Shi B. (2026). Large-Scalable Production of Sustainable, Degradable and Recyclable Bird-Nest-like Foam via Bamboo Cell Reassembly and Direct Atmospheric Drying. Ind. Crops Prod..

[42] Gupta B. (2008). Textile Fiber Morphology, Structure and Properties in Relation to Friction. Friction in Textile Materials.

[43] Sekar V., Cantwell W.J., Liao K., Berton B., Jacquart P.-M., Abu Al-Rub R.K. (2024). Additively Manufactured Metamaterials for Acoustic Absorption: A Review. Virtual Phys. Prototyp..

[44] Li Z., Zhai W., Li X., Yu X., Guo Z., Wang Z. (2022). Additively Manufactured Dual-Functional Metamaterials with Customisable Mechanical and Sound-Absorbing Properties. Virtual Phys. Prototyp..

[45] Cao L., Fu Q., Si Y., Ding B., Yu J. (2018). Porous Materials for Sound Absorption. Compos. Commun..

[46] Shen L., Zhang H., Lei Y., Chen Y., Liang M., Zou H. (2021). Hierarchical Pore Structure Based on Cellulose Nanofiber/Melamine Composite Foam with Enhanced Sound Absorption Performance. Carbohydr. Polym..

[47] Shah A.K., Jain A. (2024). Microstructure and Mechanical Properties of Filament and Fused Deposition Modelling Printed Polylactic-Acid and Carbon-Fiber Reinforced Polylactic-Acid. J. Reinf. Plast. Compos..

[48] Murugan D., Varughese S., Swaminathan T. (2006). Recycled Polyolefin-Based Plastic Wastes for Sound Absorption. Polym. Plast. Technol. Eng..

[49] Khalili Z., Sheikholeslami M. (2023). Investigation of Innovative Cooling System for Photovoltaic Solar Unit in Existence of Thermoelectric Layer Utilizing Hybrid Nanomaterial and Y-Shaped Fins. Sustain. Cities Soc..

[50] Gan Z., Qi R., Chen B., Tu W., Liao M. (2025). Ti_3_C_2_T_x_-Based Cross-Scale Laminated Structural Structures: Enabling Sub-Wavelength Impedance Modulation and Underwater Broadband Sound Absorption. Small.

[51] Allard J.F., Atalla N. (2009). Propagation of Sound in Porous Media.

[52] Panneton R., Olny X. (2006). Acoustical Determination of the Parameters Governing Viscous Dissipation in Porous Media. J. Acoust. Soc. Am..

[53] Yang Y., Liu C., Shi F., Yang Y., Liu L. (2025). Influence of Thermal and Viscous Effects on Acoustic Energy Transmission Characteristics in Microcolumn Arrays. Appl. Therm. Eng..

[54] Olny X., Panneton R. (2008). Acoustical Determination of the Parameters Governing Thermal Dissipation in Porous Media. J. Acoust. Soc. Am..

[55] Langfeldt F., Hoppen H., Gleine W. (2019). Resonance Frequencies and Sound Absorption of Helmholtz Resonators with Multiple Necks. Appl. Acoust..

[56] Kim B., Kim S., Park Y., Mieremet M., Yang H., Baek J., Choi S. (2021). Development of a Slit-Type Soundproof Panel for a Reduction in Wind Load and Low-Frequency Noise with Helmholtz Resonators. Appl. Sci..

[57] Griffin S., Lane S.A., Huybrechts S. (2001). Coupled Helmholtz Resonators for Acoustic Attenuation. J. Vib. Acoust..

[58] Qiu X. (2016). Principles of Sound Absorbers. Acoustic Textiles.

[59] Arenas C., Leiva C., Vilches L.F., Cifuentes H., Rodríguez-Galán M. (2015). Technical Specifications for Highway Noise Barriers Made of Coal Bottom Ash-Based Sound Absorbing Concrete. Constr. Build. Mater..

[60] Petrone G., D’Alessandro V., Franco F., De Rosa S. (2015). Damping Evaluation on Eco-Friendly Sandwich Panels through Reverberation Time (*RT*_60_) Measurements. J. Vib. Control.

[61] Lee S.-M., Park C.-J., Haan C.-H. (2022). Investigation of the Appropriate Reverberation Time in Learning Spaces for Elderly People Using Speech Intelligibility Tests. Buildings.

[62] Abdullah R., Ismail S., Nik Dzulkefli N. (2020). Potential Acoustic Treatment Analysis Using Sabine Formula in Unoccupied Classroom. J. Phys. Conf. Ser..

[63] Cox T., D’Antonio P. (2016). Acoustic Absorbers and Diffusers.

[64] Davern W.A. (1977). Perforated Facings Backed with Porous Materials as Sound Absorbers—An Experimental Study. Appl. Acoust..

[65] Jordan V.L. (1947). The Application of Helmholtz Resonators to Sound-Absorbing Structures. J. Acoust. Soc. Am..

[66] Frommhold W., Fuchs H.V., Sheng S. (1994). Acoustic Performance of Membrane Absorbers. J. Sound Vib..

[67] Qie Z., Rabbani A., Liang Y., Sun F., Behnsen J., Wang Y., Wang S., Zhang Y., Alhassawi H., Gao J. (2022). Multiscale Investigation of Pore Network Heterogeneity and Permeability of Fluid Catalytic Cracking (FCC) Particles. Chem. Eng. J..

[68] Rahimabady M., Statharas E.C., Yao K., Sharifzadeh Mirshekarloo M., Chen S., Tay F.E.H. (2017). Hybrid Local Piezoelectric and Conductive Functions for High Performance Airborne Sound Absorption. Appl. Phys. Lett..

[69] Lee Y.Y., Lee E.W.M., Ng C.F. (2005). Sound Absorption of a Finite Flexible Micro-Perforated Panel Backed by an Air Cavity. J. Sound Vib..

[70] Mohammadi M., Ishak M.R., Sultan M.T.H., Zainudin E.S. (2025). A Comprehensive Review of Factors Influencing the Sound Absorption Properties of Micro-Perforated Panel Structures. J. Vib. Eng. Technol..

[71] Bujoreanu C., Nedeff F., Benchea M., Agop M. (2017). Experimental and Theoretical Considerations on Sound Absorption Performance of Waste Materials Including the Effect of Backing Plates. Appl. Acoust..

[72] Dunne R., Desai D., Sadiku R. (2017). A Review of the Factors That Influence Sound Absorption and the Available Empirical Models for Fibrous Materials. Acoust. Aust..

[73] Egab L., Wang X., Fard M. (2014). Acoustical Characterisation of Porous Sound Absorbing Materials: A Review. Int. J. Veh. Noise Vib..

[74] Rey R.D., Alba J., Arenas J.P., Ramis J. (2013). Technical Notes: Evaluation of Two Alternative Procedures for Measuring Airflow Resistance of Sound Absorbing Materials. Arch. Acoust..

[75] Champoux Y., Allard J.-F. (1991). Dynamic Tortuosity and Bulk Modulus in Air-Saturated Porous Media. J. Appl. Phys..

[76] Mamtaz H., Fouladi M.H., Al-Atabi M., Narayana Namasivayam S. (2016). Acoustic Absorption of Natural Fiber Composites. J. Eng..

[77] Sevostianov I. (2022). Characterization of Physical Properties of a Porous Material in Terms of Tortuosity of the Porous Space: A Review. Advanced Materials Modelling for Mechanical, Medical and Biological Applications.

[78] Nick A., Becker U., Thoma W. (2002). Improved Acoustic Behavior of Interior Parts of Renewable Resources in the Automotive Industry. J. Polym. Environ..

[79] Taban E., Valipour F., Abdi D.D., Amininasab S. (2021). Mathematical and Experimental Investigation of Sound Absorption Behavior of Sustainable Kenaf Fiber at Low Frequency. Int. J. Environ. Sci. Technol..

[80] Lim Z.Y., Putra A., Nor M.J.M., Yaakob M.Y. (2018). Sound Absorption Performance of Natural Kenaf Fibres. Appl. Acoust..

[81] Zhou N., Zhao M., Xu B., Xie L., Liu D., Qu L., Han W. (2023). Effects of Fiber Aspect Ratio and Fabrication Temperature on the Microstructure and Mechanical Properties of Elastic Fibrous Porous Ceramics by Press-Filtration Method. Ceram. Int..

[82] Bahiraei F., SheikhMozafari M.J., Soltani P., Mirzaei R., Taban E. (2026). Acoustic and Thermal Performance of Sustainable Pine Needle Fiber Panels with Integrated Micro-Perforated Panels for Eco-Friendly Building Applications. Measurement.

[83] Fatima S., Mohanty A.R. (2011). Acoustical and Fire-Retardant Properties of Jute Composite Materials. Appl. Acoust..

[84] Chen W., Zhang S., He F., Lu W., Xv H. (2019). Porosity and Surface Chemistry Development and Thermal Degradation of Textile Waste Jute during Recycling as Activated Carbon. J. Mater. Cycles Waste Manag..

[85] Glé P., Gourdon E., Arnaud L. (2012). Modelling of the Acoustical Properties of Hemp Particles. Constr. Build. Mater..

[86] Glé P., Gourdon E., Arnaud L., Horoshenkov K.-V., Khan A. (2013). The Effect of Particle Shape and Size Distribution on the Acoustical Properties of Mixtures of Hemp Particles. J. Acoust. Soc. Am..

[87] Siouta L., Apostolopoulou M., Bakolas A. (2024). Natural Fibers in Composite Materials for Sustainable Building: A State-of-the-Art Review on Treated Hemp Fibers and Hurds in Mortars. Sustainability.

[88] Bhingare N.H., Prakash S. (2021). An Experimental and Theoretical Investigation of Coconut Coir Material for Sound Absorption Characteristics. Mater. Today Proc..

[89] Taban E., Khavanin A., Faridan M., Samaei S.E., Samimi K., Rashidi R. (2020). Comparison of Acoustic Absorption Characteristics of Coir and Date Palm Fibers: Experimental and Analytical Study of Green Composites. Int. J. Environ. Sci. Technol..

[90] Silva C.C.B.d., Terashima F.J.H., Barbieri N., Lima K.F.d. (2019). Sound Absorption Coefficient Assessment of Sisal, Coconut Husk and Sugar Cane Fibers for Low Frequencies Based on Three Different Methods. Appl. Acoust..

[91] Vigneshwaran K., Venkateshwaran N., Shanthi R., Kannan G., Kumar B.R., Shanmugam V., Das O. (2024). The Acoustic Properties of FDM Printed Wood/PLA-Based Composites. Compos. Part C Open Access.

[92] Ahmed R., Manik K.H., Nath A., Shohag J.R., Mim J.J., Hossain N. (2025). Recent Advances in Sustainable Natural Fiber Composites: Environmental Benefits, Applications, and Future Prospects. Mater. Today Sustain..

[93] Hassan T., Jamshaid H., Mishra R., Khan M.Q., Petru M., Tichy M., Muller M. (2021). Factors Affecting Acoustic Properties of Natural-Fiber-Based Materials and Composites: A Review. Textiles.

[94] Nandanwar A., Kiran M.C., Varadarajulu K.C. (2017). Influence of Density on Sound Absorption Coefficient of Fibre Board. Open J. Acoust..

[95] Chandroji Rao K.M., Sheshagiri M.B., Ramamoorthy R.V., Amran M., Nandanwar A., Vijayakumar P., Avudaiappan S., Guindos P. (2024). Effect of Density on Acoustic and Thermal Properties of Low-Density Particle Boards Made from Agro-Residues: Towards Sustainable Material Solutions. Bioresources.

[96] Balador Z., Gjerde M., Isaacs N., Imani M. (2018). Thermal and Acoustic Building Insulations from Agricultural Wastes. Handbook of Ecomaterials.

[97] Maderuelo-Sanz R., García-Cobos F.J., Sánchez-Delgado F.J., Mota-López M.I., Meneses-Rodríguez J.M., Romero-Casado A., Acedo-Fuentes P., López-Ramos L. (2022). Mechanical, Thermal and Acoustical Evaluation of Biocomposites Made of Agricultural Waste for Ceiling Tiles. Appl. Acoust..

[98] Beheshti M.H., Firoozi A., Jafarizaveh M., Tabrizi A. (2023). Acoustical and Thermal Characterization of Insulating Materials Made from Wool and Sugarcane Bagasse. J. Nat. Fibers.

[99] Poeiras A.P., Silva M.E., Günther B., Vogel C., Surový P., de Almeida Ribeiro N. (2021). Cork Influenced by a Specific Water Regime—Macro and Microstructure Characterization: The First Approach. Wood Sci. Technol..

[100] Manh N.C., Hung N.T.Q., Oanh H.T.N., Mori Y. (2026). Design Constraints Governing Household-Scale Rice-Straw Biochar Systems: The Role of Feedstock Densification and Particle-Size Selection. Case Stud. Chem. Environ. Eng..

[101] Liang M., Wu H., Yang T., Liu W., Ibarias M., Marburg S., Sánchez-Dehesa J. (2025). Metasurfaces with Low-Frequency Broadband High Absorption Based on Thermoviscous Theoretical Model and Acoustic Bands under Wide-Angle Incidence Waves. Thin-Walled Struct..

[102] Riseh R.S., Vazvani M.G., Hassanisaadi M., Thakur V.K. (2024). Agricultural Wastes: A Practical and Potential Source for the Isolation and Preparation of Cellulose and Application in Agriculture and Different Industries. Ind. Crops Prod..

[103] Kolya H., Kang C.-W. (2024). Sugar Maple (*Acer saccharum*) Waste Leaves as a Renewable Resource for Sound Absorption: An Eco-Conscious Approach. J. Environ. Chem. Eng..

[104] Zohra Mesli I.F., Hachemi H., Benosman A.S., Taleb O., Houti F.B., Seladji C., Mouaissa M.S. (2026). Enhancing Thermal Insulation and Humidity Control in Composite Plaster Mortars with Olive Pomace Waste: Experimental and Numerical Study. Energy Build..

[105] Romano, Grammatikos S., Riley M., Bras A. (2021). Analysis of Dynamic Moisture Movement within Bio-Based Earth Mortars. Constr. Build. Mater..

[106] Sun W., Tajvidi M., Howell C., Hunt C.G. (2022). Insight into Mycelium-Lignocellulosic Bio-Composites: Essential Factors and Properties. Compos. Part A Appl. Sci. Manuf..

[107] Girometta C., Picco A.M., Baiguera R.M., Dondi D., Babbini S., Cartabia M., Pellegrini M., Savino E. (2019). Physico-Mechanical and Thermodynamic Properties of Mycelium-Based Biocomposites: A Review. Sustainability.

[108] Al-Qahtani S., Koç M., Isaifan R.J. (2023). Mycelium-Based Thermal Insulation for Domestic Cooling Footprint Reduction: A Review. Sustainability.

[109] Parhizi Z., Dearnaley J., Kauter K., Mikkelsen D., Pal P., Shelley T., Burey P. (2025). The Fungus Among Us: Innovations and Applications of Mycelium-Based Composites. J. Fungi.

[110] Dias P.P., Jayasinghe L.B., Waldmann D. (2021). Investigation of Mycelium-Miscanthus Composites as Building Insulation Material. Results Mater..

[111] Majib N.M., Yaacob N.D., Ting S.S., Rohaizad N.M., Azizul Rashidi A.M. (2025). Fungal Mycelium-Based Biofoam Composite: A Review in Growth, Properties and Application. Prog. Rubber Plast. Recycl. Technol..

[112] Alaneme K.K., Anaele J.U., Oke T.M., Kareem S.A., Adediran M., Ajibuwa O.A., Anabaranze Y.O. (2023). Mycelium Based Composites: A Review of Their Bio-Fabrication Procedures, Material Properties and Potential for Green Building and Construction Applications. Alex. Eng. J..

[113] Deng T., Garg V., Bradley M.S.A. (2025). Review of Material-Handling Challenges in Energy Production from Biomass and Other Solid Waste Materials. Energies.

[114] Le Ferrand H. (2024). Critical Review of Mycelium-Bound Product Development to Identify Barriers to Entry and Paths to Overcome Them. J. Clean. Prod..

[115] Oldham D.J., Egan C.A., Cookson R.D. (2011). Sustainable Acoustic Absorbers from the Biomass. Appl. Acoust..

[116] Drozdov A.D., de Claville Christiansen J. (2020). The Effect of Porosity on Elastic Moduli of Polymer Foams. J. Appl. Polym. Sci..

[117] Pelletier M.G., Holt G.A., Wanjura J.D., Bayer E., McIntyre G. (2013). An Evaluation Study of Mycelium Based Acoustic Absorbers Grown on Agricultural By-Product Substrates. Ind. Crops Prod..

[118] Jones M., Mautner A., Luenco S., Bismarck A., John S. (2020). Engineered Mycelium Composite Construction Materials from Fungal Biorefineries: A Critical Review. Mater. Des..

[119] Walter N., Gürsoy B. (2022). A Study on the Sound Absorption Properties of Mycelium-Based Composites Cultivated on Waste Paper-Based Substrates. Biomimetics.

[120] Tang X., Yan X. (2017). Acoustic Energy Absorption Properties of Fibrous Materials: A Review. Compos. Part A Appl. Sci. Manuf..

[121] Baseri S. (2024). Environmentally Sound Recycling of Agricultural Waste: A Sustainable Approach to Develop Bio-Functional Art Textile. J. Environ. Manag..

[122] Allard J.-F., Champoux Y. (1992). New Empirical Equations for Sound Propagation in Rigid Frame Fibrous Materials. J. Acoust. Soc. Am..

[123] Faruk O., Bledzki A.K., Fink H.-P., Sain M. (2012). Biocomposites Reinforced with Natural Fibers: 2000–2010. Prog. Polym. Sci..

[124] Tanaka M., Hojo M., Hobbiebrunken T., Ochiai S., Hirosawa Y., Fujita K., Sawada Y. (2005). Influence of Non-Uniform Fiber Arrangement on Tensile Fracture Behavior of Unidirectional Fiber/Epoxy Model Composites. Compos. Interfaces.

[125] Bourbigot S., Flambard X. (2002). Heat Resistance and Flammability of High Performance Fibres: A Review. Fire Mater..

[126] Yang Y., Haurie L., Wang D.-Y. (2022). Bio-Based Materials for Fire-Retardant Application in Construction Products: A Review. J. Therm. Anal. Calorim..

[127] Liu Q., Yan Y., Meng L., Zhang Z., Zhou P. (2022). Influence of Airflow Disturbance on the Uniformity of Spin Coating Film Thickness on Large Area Rectangular Substrates. Coatings.

[128] Copenhaver K., Smith T., Armstrong K., Kamath D., Rencheck M., Bhagia S., Korey M., Lamm M., Ozcan S. (2023). Recyclability of Additively Manufactured Bio-Based Composites. Compos. B Eng..

[129] Ricciardi P., Belloni E., Cotana F. (2014). Innovative Panels with Recycled Materials: Thermal and Acoustic Performance and Life Cycle Assessment. Appl. Energy.

[130] Siltumens K. (2025). Acoustic Performance of Panels Made from Recycled Polyester Fiber Derived from PET Bottles. IOP Conf. Ser. Earth Environ. Sci..

[131] Sharma D., Jee A.A., Kumari S., Kumar A., Kumar S., Bansal T., Kumar P. (2025). Characteristics of Concrete Using Recycled Rubber. Binding Materials for Sustainable Construction.

[132] Enking J., Becker A., Schu G., Gausmann M., Cucurachi S., Tukker A., Gries T. (2025). Recycling Processes of Polyester-Containing Textile Waste–A Review. Resour. Conserv. Recycl..

[133] Zare Y. (2013). Recent Progress on Preparation and Properties of Nanocomposites from Recycled Polymers: A Review. Waste Manag..

[134] Fazli A., Rodrigue D. (2021). Morphological and Mechanical Properties of Thermoplastic Elastomers Based on Recycled High Density Polyethylene and Recycled Natural Rubber. Int. Polym. Process..

[135] Nimkar U. (2018). Sustainable Chemistry: A Solution to the Textile Industry in a Developing World. Curr. Opin. Green Sustain. Chem..

[136] Imtiazuddin S., Mumtaz M., Mallick K.A. (2012). Pollutants of Wastewater Characteristics in Textile Industries. J. Basic Appl. Sci..

[137] Candido R.G. (2021). Recycling of Textiles and Its Economic Aspects. Fundamentals of Natural Fibres and Textiles.

[138] Abtew M.A., Atalie D., Dejene B.K. (2025). Recycling of Cotton Textile Waste: Technological Process, Applications, and Sustainability within a Circular Economy. J. Ind. Text..

[139] Ferreira Junior J.C., Triki E., Doutres O., Demarquette N.R., Hof L.A. (2025). Recyclable Polyester Textile Waste-Based Composites for Building Applications in a Circular Economy Framework. J. Clean. Prod..

[140] Sakthivel S., Senthil Kumar S., Melese B., Mekonnen S., Solomon E., Edae A., Abedom F., Gedilu M. (2021). Development of Nonwoven Composites from Recycled Cotton/Polyester Apparel Waste Materials for Sound Absorbing and Insulating Properties. Appl. Acoust..

[141] Zhou X., Zheng F., Li H., Lu C. (2010). An Environment-Friendly Thermal Insulation Material from Cotton Stalk Fibers. Energy Build..

[142] Miskinis K., Dikavicius V., Buska A., Banionis K. (2018). Influence of EPS, Mineral Wool and Plaster Layers on Sound and Thermal Insulation of a Wall: A Case Study. Appl. Acoust..

[143] Sakthivel S., Senthil Kumar B. (2021). Studies on Influence of Bonding Methods on Sound Absorption Characteristic of Polyester/Cotton Recycled Nonwoven Fabrics. Appl. Acoust..

[144] Su J., Zheng L., Deng Z. (2019). Study on Acoustic Properties at Normal Incidence of Three-Multilayer Composite Made of Glass Wool, Glue and Polyurethane Foam. Appl. Acoust..

[145] Mu B., Yu X., Yang Y. (2023). Sustainable and Green Process for Recycling Waste Wool Textiles into High-Quality Protein Fibers on a Pilot Scale. Resour. Conserv. Recycl..

[146] Yang Y., Li C., Zou R., Liu X., Wei S., Cheng K., Song L., Yang T. (2026). Study on the Influence of Pore Structure on the Foam Concrete towards Sound Absorption Enhancement. Constr. Build. Mater..

[147] Leofanti G., Padovan M., Tozzola G., Venturelli B. (1998). Surface Area and Pore Texture of Catalysts. Catal. Today.

[148] Seifali Abbas-Abadi M., Tomme B., Goshayeshi B., Mynko O., Wang Y., Roy S., Kumar R., Baruah B., De Clerck K., De Meester S. (2025). Advancing Textile Waste Recycling: Challenges and Opportunities Across Polymer and Non-Polymer Fiber Types. Polymers.

[149] Anas M.S., Faheem M., Mikucioniene D. (2025). Challenges and Limitations in Recycling of Post-Consumer Cotton Denim Waste into New Textiles. J. Nat. Fibers.

[150] Cavalli M.C., Zaumanis M., Mazza E., Partl M.N., Poulikakos L.D. (2018). Effect of Ageing on the Mechanical and Chemical Properties of Binder from RAP Treated with Bio-Based Rejuvenators. Compos. B Eng..

[151] Vaishali M., Gopal S., Sreeram K.J. (2024). A Facile Approach towards Recycling of Polyurethane Coated PET Fabrics. RSC Sustain..

[152] Pillich M., Schilling J., Bosetti L., Bardow A. (2024). What to Do with Polyurethane Waste? The Environmental Potential of Chemically Recycling Polyurethane Rigid Foam. Green Chem..

[153] Srivastav R.S., More A.P. (2026). Advanced Techniques and Mechanistic Insights for High Efficiency Recovery of Polymers Recycling. J. Environ. Manag..

[154] Shahani F., Soltani P., Zarrebini M. (2014). The Analysis of Acoustic Characteristics and Sound Absorption Coefficient of Needle Punched Nonwoven Fabrics. J. Eng. Fiber. Fabr..

[155] Stein R.S. (1992). Polymer Recycling: Opportunities and Limitations. Proc. Natl. Acad. Sci. USA.

[156] Chen K., Wang D., Du J., Cheng Q., Zhang L., Xia W., Wang Y., Zhou H. (2025). Excellent Broadband Sound Absorption in Composites Manufactured by Embedding Piezoelectric Polymer Aerogels in Porous Ceramics. J. Colloid Interface Sci..

[157] Gama N.V., Ferreira A., Barros-Timmons A. (2018). Polyurethane Foams: Past, Present, and Future. Materials.

[158] Duan Y., Chen X., Xia H., Liu Y., You F., Jiang X., Ren L., Zhou D. (2025). Fabrication, Structural Regulation and Future Applications of Acoustic Polymer Materials: A Review. Appl. Mater. Today.

[159] Dewang Y., Sharma V., Singla Y.K. (2025). A Critical Review of Waste Tire Pyrolysis for Diesel Engines: Technologies, Challenges, and Future Prospects. Sustain. Mater. Technol..

[160] Yang Y.-L., Zhang T., Chen Y., Wang C.-J., Cai G.-J. (2025). A Comprehensive Review on Application of Scrap Tire Rubber for Sustainable Thermal Insulation Material in Civil Engineering. Renew. Sustain. Energy Rev..

[161] Thai Q.B., Chong R.O., Nguyen P.T.T., Le D.K., Le P.K., Phan-Thien N., Duong H.M. (2020). Recycling of Waste Tire Fibers into Advanced Aerogels for Thermal Insulation and Sound Absorption Applications. J. Environ. Chem. Eng..

[162] Yang J., Gao X., Xu J., Lacidogna G., Shao J., Zhu H., Liu C., Ye C. (2024). Insights into the Fracture Properties of Recycled Ceramic and Rubber Composite Cement-Based Materials: Fracture Mechanics, Acoustic Emission, and Digital Image Correlation. Constr. Build. Mater..

[163] Shadfar A., Nani M., Shirinabadi R., Hosseini S.A. (2025). The Feasibility of Constructing Rubber Concrete Pavement Reinforced with Recycled and Industrial Steel Fibers. Results Mater..

[164] Zhang Z.X., Zhang T., Wang D., Zhang X., Xin Z., Prakashan K. (2018). Physicomechanical, Friction, and Abrasion Properties of EVA/PU Blend Foams Foamed by Supercritical Nitrogen. Polym. Eng. Sci..

[165] Zhang P., Wang Y., Liu B., Xiao J. (2026). Effect of Load History on the Material Damping Evolution of Recycled Aggregate Concrete: Based on Viscoelastic Interconversion of Short-Term Creep and Damping. Constr. Build. Mater..

[166] Myhre M., Saiwari S., Dierkes W., Noordermeer J. (2012). Rubber Recycling: Chemistry, Processing, and Applications. Rubber Chem. Technol..

[167] Swift M.J., Bris P., Horoshenkov K.V. (1999). Acoustic Absorption in Re-Cycled Rubber Granulate. Appl. Acoust..

[168] Cao L., Si Y., Wu Y., Wang X., Yu J., Ding B. (2019). Ultralight, Superelastic and Bendable Lashing-Structured Nanofibrous Aerogels for Effective Sound Absorption. Nanoscale.

[169] Ziari H., Divandari H., Seyed Ali Akbar S.M., Hosseinian S.M. (2021). Investigation of the Effect of Crumb Rubber Powder and Warm Additives on Moisture Resistance of SMA Mixtures. Adv. Civ. Eng..

[170] Ince C., Shehata B.M.H., Derogar S., Ball R.J. (2022). Towards the Development of Sustainable Concrete Incorporating Waste Tyre Rubbers: A Long-Term Study of Physical, Mechanical & Durability Properties and Environmental Impact. J. Clean. Prod..

[171] Vasina M., Monkova K., Monka P.P., Kozak D., Tkac J. (2020). Study of the Sound Absorption Properties of 3D-Printed Open-Porous ABS Material Structures. Polymers.

[172] Bhuvaneswari V., Devarajan B., Arulmurugan B., Mahendran R., Rajkumar S., Sharma S., Mausam K., Li C., Eldin E.T. (2022). A Critical Review on Hygrothermal and Sound Absorption Behavior of Natural-Fiber-Reinforced Polymer Composites. Polymers.

[173] Sola A., Trinchi A. (2023). Recycling as a Key Enabler for Sustainable Additive Manufacturing of Polymer Composites: A Critical Perspective on Fused Filament Fabrication. Polymers.

[174] Rey R.d., Alba J., Arenas J.P., Sanchis V.J. (2012). An Empirical Modelling of Porous Sound Absorbing Materials Made of Recycled Foam. Appl. Acoust..

[175] Puguan J.M.C., Pornea A.G.M., Ruello J.L.A., Kim H. (2022). Double-Porous PET Waste-Derived Nanofibrous Aerogel for Effective Broadband Acoustic Absorption and Transmission. ACS Appl. Polym. Mater..

[176] Mukherjee C., Varghese D., Krishna J.S., Boominathan T., Rakeshkumar R., Dineshkumar S., Brahmananda Rao C.V.S., Sivaramakrishna A. (2023). Recent Advances in Biodegradable Polymers—Properties, Applications and Future Prospects. Eur. Polym. J..

[177] Rabearivony A.F., Murad N.M. (2026). Sustainable Acoustic Metamaterials from Recycled Plastic Bottles: A Comprehensive Review for Circular Noise Control in the Built Environment. Sustain. Mater. Technol..

[178] Chang B.P., Gupta A., Muthuraj R., Mekonnen T.H. (2021). Bioresourced Fillers for Rubber Composite Sustainability: Current Development and Future Opportunities. Green Chem..

[179] Sienkiewicz M., Janik H., Borzędowska-Labuda K., Kucińska-Lipka J. (2017). Environmentally Friendly Polymer-Rubber Composites Obtained from Waste Tyres: A Review. J. Clean. Prod..

[180] Gruszczyński M., Kowalska-Koczwara A., Tatara T. (2025). Effect of Rubber Granulate Content on the Compressive Strength of Concrete for Industrial Vibration-Isolating Floors. Materials.

[181] Sona S., Sangeetha S.P. (2025). Eco-Friendly Alternative Activators Derived from Industrial Wastes for the Sustainable Production of Two-Part Geopolymer Concrete at Low Cost. Constr. Build. Mater..

[182] Yang Y., Wang B., Yuan Q., Huang D., Peng H. (2023). Characterization, Factors, and Fractal Dimension of Pore Structure of Fly Ash-Based Geopolymers. J. Mater. Res. Technol..

[183] Su L., Fu G., Liang B., Sun Q., Zhang X. (2022). Mechanical Properties and Microstructure Evaluation of Fly Ash—Slag Geopolymer Foaming Materials. Ceram. Int..

[184] Singh N. (2018). Fly Ash-Based Geopolymer Binder: A Future Construction Material. Minerals.

[185] Novais R.M., Carvalheiras J., Senff L., Lacasta A.M., Cantalapiedra I.R., Giro-Paloma J., Seabra M.P., Labrincha J.A. (2020). Multifunctional Cork—Alkali-Activated Fly Ash Composites: A Sustainable Material to Enhance Buildings’ Energy and Acoustic Performance. Energy Build..

[186] Emarah D.A. (2025). Multivariate Predictive Modeling of Compressive Strength in Ground Granulated Blast Furnace Slag/Fly Ash-Based Alkali-Activated Concrete. Clean. Eng. Technol..

[187] Nodehi M. (2021). A Comparative Review on Foam-Based versus Lightweight Aggregate-Based Alkali-Activated Materials and Geopolymer. Innov. Infrastruct. Solut..

[188] Alves Z., Carvalheiras J., Senff L., Lacasta A.M., Cantalapiedra I.R., Labrincha J.A., Novais R.M. (2024). A Comparison between the Use of Cork and Synthetic Aggregates in the Production of Geopolymer Composites. Constr. Build. Mater..

[189] Muthusamy M., Vanitha N., Karuppaiyan J., Jeyalakshmi R. (2026). Evaluation of Thermal and Acoustic Resistance of Alkali-Activated Coal Fly Ash Blended with Slag for Porous Geopolymer Binder Using Response Surface Methodology Approach. Int. J. Coal Prep. Util..

[190] Fan J., Zhang L., Wei S., Zhang Z., Choi S.-K., Song B., Shi Y. (2021). A Review of Additive Manufacturing of Metamaterials and Developing Trends. Mater. Today.

[191] Afshar M., Anaraki A.P., Montazerian H., Kadkhodapour J. (2016). Additive Manufacturing and Mechanical Characterization of Graded Porosity Scaffolds Designed Based on Triply Periodic Minimal Surface Architectures. J. Mech. Behav. Biomed. Mater..

[192] Islam M.A., Mobarak M.H., Rimon M.I.H., Al Mahmud M.Z., Ghosh J., Ahmed M.M.S., Hossain N. (2024). Additive Manufacturing in Polymer Research: Advances, Synthesis, and Applications. Polym. Test..

[193] Ceretti D.V.A., Edeleva M., Cardon L., D’hooge D.R. (2023). Molecular Pathways for Polymer Degradation during Conventional Processing, Additive Manufacturing, and Mechanical Recycling. Molecules.

[194] Marabello G., Chairi M., Di Bella G. (2024). Optimising Additive Manufacturing to Produce PLA Sandwich Structures by Varying Cell Type and Infill: Effect on Flexural Properties. J. Compos. Sci..

[195] Maskery I., Sturm L., Aremu A.O., Panesar A., Williams C.B., Tuck C.J., Wildman R.D., Ashcroft I.A., Hague R.J.M. (2018). Insights into the Mechanical Properties of Several Triply Periodic Minimal Surface Lattice Structures Made by Polymer Additive Manufacturing. Polymer.

[196] Li X., Chua J.W., Yu X., Li Z., Zhao M., Wang Z., Zhai W. (2024). 3D-Printed Lattice Structures for Sound Absorption: Current Progress, Mechanisms and Models, Structural-Property Relationships, and Future Outlook. Adv. Sci..

[197] Li X., Qu P., Kong H., Lei Y., Guo A., Wang S., Wan Y., Takahashi J. (2024). Enhanced Mechanical Properties of Sandwich Panels via Integrated 3D Printing of Continuous Fiber Face Sheet and TPMS Core. Thin-Walled Struct..

[198] Kafle A., Luis E., Silwal R., Pan H.M., Shrestha P.L., Bastola A.K. (2021). 3D/4D Printing of Polymers: Fused Deposition Modelling (FDM), Selective Laser Sintering (SLS), and Stereolithography (SLA). Polymers.

[199] Chacón J.M., Caminero M.A., García-Plaza E., Núñez P.J. (2017). Additive Manufacturing of PLA Structures Using Fused Deposition Modelling: Effect of Process Parameters on Mechanical Properties and Their Optimal Selection. Mater. Des..

[200] Striani R., Fico D., Rizzo D., Ferrari F., Lionetto F., Esposito Corcione C. (2025). Polymer-Based Materials: Focus on Sustainability and Recycled Materials for 3D Printing Application. Comprehensive Green Materials.

[201] Sardinha M., Ferreira L., Diogo H., Ramos T.R.P., Reis L., Vaz M.F. (2025). Material Extrusion of TPU: Thermal Characterization and Effects of Infill and Extrusion Temperature on Voids, Tensile Strength and Compressive Properties. Rapid Prototyp. J..

[202] Fritz F., Haefele S., Traut A., Eckerle M. (2013). Manufacturing of Optimized Venturi Nozzles Based on Technical-Economic Analysis. Re-Engineering Manufacturing for Sustainability.

[203] Huang J., Qin Q., Wang J. (2020). A Review of Stereolithography: Processes and Systems. Processes.

[204] Venuvinod P.K., Ma W. (2004). Selective Laser Sintering (SLS). Rapid Prototyping.

[205] Chouhan G., Bidare P., Bala Murali G. (2024). Triply Periodic Minimal Surface Based Lattices for Acoustic Performance. Noise Vib. Worldw..

[206] Tsioukas V., Pikridas C., Karolos I.-A. (2020). Challenges, Opportunities, and Limitations in 3D Printing. 3D Printing: Applications in Medicine and Surgery.

[207] Gauthé M., Lorrette C., Chaffron L., Calmé S., Tonnellier X., Rodolfo J., Sortais Y. (2025). Fused Filament Fabrication of Silicon Carbide Parts: A Strategy for Producing High-Strength Components. J. Eur. Ceram. Soc..

[208] Maidin S., Muhamad M., Pei E. (2015). Feasibility Study of Ultrasonic Frequency Application on fdm to Improve Parts Surface Finish. J. Teknol..

[209] Xu S., Ahmed S., Momin M., Hossain A., Zhou T. (2023). Unleashing the Potential of 3D Printing Soft Materials. Device.

[210] Sirivuri K.K., Sekar V., Cantwell W.J., Liao K., Berton B., Ravaud N., Jacquart P.-M., Abu Al-Rub R.K. (2025). Computational Study of Sound Absorption in TPMS Lattice Materials Using a Thermoviscous Model. J. Build. Eng..

[211] Sung G., Kim J.W., Kim J.H. (2016). Fabrication of Polyurethane Composite Foams with Magnesium Hydroxide Filler for Improved Sound Absorption. J. Ind. Eng. Chem..

[212] Gama N., Ferreira A., Barros-Timmons A. (2019). 3D Printed Cork/Polyurethane Composite Foams. Mater. Des..

[213] Liu Z., Zhan J., Fard M., Davy J.L. (2017). Acoustic Properties of Multilayer Sound Absorbers with a 3D Printed Micro-Perforated Panel. Appl. Acoust..

[214] Shafeer P.P.M., Pitchaimani J., Doddamani M. (2023). 3D Printed Thick Micro-Perforated Panel with Graded Perforation for Practical Wall Sound Absorption Applications. Acoust. Aust..

[215] Li X., Yu X., Zhai W. (2021). Additively Manufactured Deformation-Recoverable and Broadband Sound-Absorbing Microlattice Inspired by the Concept of Traditional Perforated Panels. Adv. Mater..

[216] Godakawela J., Lomte A., Sharma B. (2025). Sound Absorption in Uniform and Layered Gyroid and Diamond Triply Periodic Minimal Surface Porous Absorbers. Appl. Acoust..

[217] Singh G., Singh J., Singh N., Farina I., Colangelo F., Pandey P.M. (2024). Effect of Unit Cell Shape and Structure Volume Fraction on the Mechanical and Vibration Properties of 3D Printed Lattice Structures. J. Thermoplast. Compos. Mater..

[218] Entezari A., Wu Q., Mirkhalaf M., Lu Z., Roohani I., Li Q., Dunstan C.R., Jiang X., Zreiqat H. (2024). Unraveling the Influence of Channel Size and Shape in 3D Printed Ceramic Scaffolds on Osteogenesis. Acta Biomater..

[219] Panahi E., Braghin F., Corigliano A., Sangiuliano L., D’Alessandro L. (2026). Compacted Meta-Panel through Inhomogeneous Double Layer Micro Perforated and Coiled up Space Channels for a Broadband Sound Absorption: FEM Based Optimization and Experiments. Appl. Acoust..

[220] Yuvaraj L., Jeyanthi S., Mailan Chinnapandi L.B., Pitchaimani J. (2022). Experimental and Numerical Investigation on Sound Absorption Characteristics of 3D Printed Coupled-Cavity Integrated Passive Element Systems. J. Low Freq. Noise Vib. Act. Control.

[221] Melkisedek B.L., Emeliana Y., Dharmawan I.A. (2025). A Comparative Study of Three-Dimensional Flow Based, Geometric, and Empirical Tortuosity Models in Carbonate and Sandstone Reservoirs. Appl. Sci..

[222] Gardiner A., Daly P., Domingo-Roca R., Windmill J., Feeney A., Jackson-Camargo J. (2021). Additive Manufacture of Small-Scale Metamaterial Structures for Acoustic and Ultrasonic Applications. Micromachines.

[223] Bai H., Chen Y., Delattre B., Tomsia A.P., Ritchie R.O. (2015). Bioinspired Large-Scale Aligned Porous Materials Assembled with Dual Temperature Gradients. Sci. Adv..

[224] Kumar V., Hassen A.A., Kunc V., Nuttall D., Kircaliali A., Talabi S.I., Reynolds J., Vaughan J., Mhatre P., Smith T. (2026). Genesis of a Novel High-Rate Composite Manufacturing Process Using Large-Scale Additive Manufacturing—Compression Molding (AM-CM) System: Possibilities and Limitations∗. Compos. B Eng..

[225] Li X., Liu B., Wu Q. (2022). Enhanced Low-Frequency Sound Absorption of a Porous Layer Mosaicked with Perforated Resonator. Polymers.

[226] Tamburini S., Natali M., Garbin E., Panizza M., Favaro M., Valluzzi M.R. (2017). Geopolymer Matrix for Fibre Reinforced Composites Aimed at Strengthening Masonry Structures. Constr. Build. Mater..

[227] Lingyu T., Dongpo H., Jianing Z., Hongguang W. (2021). Durability of Geopolymers and Geopolymer Concretes: A Review. Rev. Adv. Mater. Sci..

[228] Giorleo L., Basu S., Piana E. (2025). Acoustic Performances of Triply Periodic Minimal Surfaces Fabricated by Additive Manufacturing: Effects of Cell Geometry, Aspect Ratio, and Wall Thickness. Addit. Manuf..

[229] Liu C.R., Wu J.H., Lu K., Zhao Z.T., Huang Z. (2019). Acoustical Siphon Effect for Reducing the Thickness in Membrane-Type Metamaterials with Low-Frequency Broadband Absorption. Appl. Acoust..

[230] Roviello G., Occhicone A., De Gregorio E., Ricciotti L., Cioffi R., Ferone C., Tarallo O. (2025). Geopolymer-Based Composite and Hybrid Materials: The Synergistic Interaction between Components. Sustain. Mater. Technol..

[231] Feng J., Le D., Nguyen S.T., Tan Chin Nien V., Jewell D., Duong H.M. (2016). Silica cellulose Hybrid Aerogels for Thermal and Acoustic Insulation Applications. Colloids Surf. A Physicochem. Eng. Asp..

[232] Altay P. (2023). The Effect of Silica Aerogel on Thermal and Sound Absorption Insulation Properties of Epoxy Plate and Glass Fiber Fabric Epoxy Composite. J. Elastomers Plast..

[233] Banerjee D., Dutta A. (2023). Polymer Foams in Environmental Applications. Novel Polymeric Materials for Environmental Applications.

[234] Wu G., Xie P., Yang H., Dang K., Xu Y., Sain M., Turng L.-S., Yang W. (2021). A Review of Thermoplastic Polymer Foams for Functional Applications. J. Mater. Sci..

[235] Delany M.E., Bazley E.N. (1970). Acoustical Properties of Fibrous Absorbent Materials. Appl. Acoust..

[236] Hedayati R., Leeflang A.M., Zadpoor A.A. (2017). Additively Manufactured Metallic Pentamode Meta-Materials. Appl. Phys. Lett..

[237] Luo Y., Zhang T., Lin X. (2022). 3D Printed Hydrogel Scaffolds with Macro Pores and Interconnected Microchannel Networks for Tissue Engineering Vascularization. Chem. Eng. J..

[238] Liu X., Li D., He L., Gu C., Chen L., Li H., Choy Y.S. (2025). Low-Frequency Broadband Acoustic Absorption Characteristics of Honeycomb-Type Gradient Perforated Porous Acoustic Metamaterials Paired with Embedded Necks. Appl. Acoust..

[239] Krasny E., Klarić S., Korjenić A. (2017). Analysis and Comparison of Environmental Impacts and Cost of Bio-Based House versus Concrete House. J. Clean. Prod..

[240] Alam M.N., Christopher L.P. (2017). A Novel, Cost-Effective and Eco-Friendly Method for Preparation of Textile Fibers from Cellulosic Pulps. Carbohydr. Polym..

[241] Karimi N., Brear M.J., Moase W.H. (2008). Acoustic and Disturbance Energy Analysis of a Flow with Heat Communication. J. Fluid Mech..

[242] Gumanová V., Sobotová L., Dzuro T., Badida M., Moravec M. (2022). Experimental Survey of the Sound Absorption Performance of Natural Fibres in Comparison with Conventional Insulating Materials. Sustainability.

[243] Hu H., Wang Y., Wang D. (2015). On the Sound Attenuation in Fluid Due to the Thermal Diffusion and Viscous Dissipation. Phys. Lett. A.

[244] Li X., Yu X., Chua J.W., Zhai W. (2023). Harnessing Cavity Dissipation for Enhanced Sound Absorption in Helmholtz Resonance Metamaterials. Mater. Horiz..

[245] Zhang C., Li J., Hu Z., Zhu F., Huang Y. (2012). Correlation between the Acoustic and Porous Cell Morphology of Polyurethane Foam: Effect of Interconnected Porosity. Mater. Des..

[246] Pop M.A., Coșniță M., Zaharia S.-M., Chicoș L.A., Croitoru C., Roată I.C., Cătană D. (2025). Influence of the Fill Value Parameters on Acoustic and Physical–Mechanical Performance of 3D-Printed Panels. Polymers.

[247] Wang D., Xu H., Wang J., Jiang C., Zhu X., Ge Q., Gu G. (2020). Design of 3D Printed Programmable Horseshoe Lattice Structures Based on a Phase-Evolution Model. ACS Appl. Mater. Interfaces.

[248] Khalifa M., Anandhan S., Wuzella G., Lammer H., Mahendran A.R. (2020). Thermoplastic Polyurethane Composites Reinforced with Renewable and Sustainable Fillers—A Review. Polym.-Plast. Technol. Mater..

[249] Luo T., Hu Y., Zhang M., Jia P., Zhou Y. (2025). Recent Advances of Sustainable and Recyclable Polymer Materials from Renewable Resources. Resour. Chem. Mater..

[250] Ivars J., Labanieh A.R., Soulat D. (2021). Effect of the Fibre Orientation Distribution on the Mechanical and Preforming Behaviour of Nonwoven Preform Made of Recycled Carbon Fibres. Fibers.

[251] Adler P.M., Thovert J.-F. (1998). Real Porous Media: Local Geometry and Macroscopic Properties. Appl. Mech. Rev..

[252] Asdrubali F., D’Alessandro F., Schiavoni S. (2015). A Review of Unconventional Sustainable Building Insulation Materials. Sustain. Mater. Technol..

[253] Chin D.D.V.S., Yahya M.N.B., Che Din N.B., Ong P. (2018). Acoustic Properties of Biodegradable Composite Micro-Perforated Panel (BC-MPP) Made from Kenaf Fibre and Polylactic Acid (PLA). Appl. Acoust..

[254] Kim Y., Lee S., Yoon H. (2021). Fire-Safe Polymer Composites: Flame-Retardant Effect of Nanofillers. Polymers.

[255] Morsy A., El-marghany A., Rana D., Anwar H., Gepreel M.A.H., Morsy A., El-Haridi N.M., Mohamed A., Soliman A. (2024). Development of Eco-Friendly Flame-Retardant Polyurethane Using Sustainable Additives. J. Mol. Liq..

[256] Grynkiewicz-Bylina B., Rakwic B., Słomka-Słupik B. (2022). Tests of Rubber Granules Used as Artificial Turf for Football Fields in Terms of Toxicity to Human Health and the Environment. Sci. Rep..

[257] Kundu D., Barathi A., Pooja K., Surya M., Jacob S., Koley A., Samanta P., Kumar V., Chintagunta A.D., Kumar N.S.S. (2026). Green Hydrogen Pathways for a Net-Zero Future: Technologies, Circular Economy Integration, Life-Cycle Performance and Safety Dimensions. RSC Adv..

[258] Ummartyotin S., Pechyen C. (2016). Strategies for Development and Implementation of Bio-Based Materials as Effective Renewable Resources of Energy: A Comprehensive Review on Adsorbent Technology. Renew. Sustain. Energy Rev..

[259] Chen Y., Zhan B., Guo B., Yang Y., Gao P., Yu Q. (2025). Utilization of Biochar to Enhance the Sustainability of Accelerated Carbonation-Cured Cement-Based Materials: Effect of Biochar Dual-Particle-Size Gradation on Mechanical Properties and Carbon Sequestration. Constr. Build. Mater..

[260] Lucas D., Petty S.M., Keen O., Luedeka B., Schlummer M., Weber R., Yazdani R., Riise B., Rhodes J., Nightingale D. (2018). Methods of Responsibly Managing End-of-Life Foams and Plastics Containing Flame Retardants: Part II. Environ. Eng. Sci..

[261] Ibrahim H., Alam G., Amna R., Faheem A. (2026). Upcycling of Waste Plastics, End-of-Life Tire Rubber, and Bio-Derived Oils for Circular Asphalt Pavements: A Systematic Review. Green Technol. Sustain..

[262] Muhammad A., Jatoi A.S., Mazari S.A., Abro R., Mubarak N.M., Ahmed S., Shah A., Memon A.Q., Akhter F., Wahocho S.A. (2021). Recent Advances and Developments in Advanced Green Porous Nanomaterial for Sustainable Energy Storage Application. J. Porous Mater..

[263] Eickhoff R., Antusch S., Baumgärtner S., Nötzel D., Hanemann T. (2022). Feedstock Development for Material Extrusion-Based Printing of Ti6Al4V Parts. Materials.

[264] Thydal T., Pind F., Jeong C.-H., Engsig-Karup A.P. (2021). Experimental Validation and Uncertainty Quantification in Wave-Based Computational Room Acoustics. Appl. Acoust..

[265] Wang J., Li S., Yang L., Liu B., Xie S., Qi R., Zhan Y., Xia H. (2024). Graphene-Based Hybrid Fillers for Rubber Composites. Molecules.

[266] Cai C., Xin F. (2025). Pressure-Adaptive Ultra-Thin Hybrid Metamaterials for Broadband Low-Frequency Underwater Sound Absorption. Thin-Walled Struct..

